# Associations between psychosocial work factors and provider mental well-being in emergency departments: A systematic review

**DOI:** 10.1371/journal.pone.0197375

**Published:** 2018-06-04

**Authors:** Anna Schneider, Matthias Weigl

**Affiliations:** Institute and Outpatient Clinic for Occupational, Social, and Environmental Medicine, Munich University Hospital, Ludwig-Maximilians-University, Munich, Germany; University Antwerp, BELGIUM

## Abstract

**Background:**

Emergency departments (ED) are complex and dynamic work environments with various psychosocial work stressors that increase risks for providers’ well-being. Yet, no systematic review is available which synthesizes the current research base as well as quantitatively aggregates data on associations between ED work factors and provider well-being outcomes.

**Objective:**

We aimed at synthesizing the current research base on quantitative associations between psychosocial work factors (classified into patient-/ task-related, organizational, and social factors) and mental well-being of ED providers (classified into positive well-being outcomes, affective symptoms and negative psychological functioning, cognitive-behavioural outcomes, and psychosomatic health complaints).

**Methods:**

A systematic literature search in eight databases was conducted in December 2017. Original studies were extracted following a stepwise procedure and predefined inclusion criteria. A standardized assessment of methodological quality and risk of bias was conducted for each study with the Quality Assessment Tool for Quantitative Studies from the Effective Public Health Practice Project. In addition to a systematic compilation of included studies, frequency and strength of quantitative associations were synthesized by means of harvest plots. Subgroup analyses for ED physicians and nurses were conducted.

**Results:**

N = 1956 records were retrieved. After removal of duplicates, 1473 records were screened for titles and abstracts. 199 studies were eligible for full-text review. Finally, 39 original studies were included whereof 37 reported cross-sectional surveys. Concerning the methodological quality of included studies, the majority was evaluated as weak to moderate with considerable risk of bias. Most frequently surveyed provider outcomes were affective symptoms (e.g., burnout) and positive well-being outcomes (e.g., job satisfaction). 367 univariate associations and 370 multivariate associations were extracted with the majority being weak to moderate. Strong associations were mostly reported for social and organizational work factors.

**Conclusions:**

To the best of our knowledge, this review is the first to provide a quantitative summary of the research base on associations of psychosocial ED work factors and provider well-being. Conclusive results reveal that peer support, well-designed organizational structures, and employee reward systems balance the negative impact of adverse work factors on ED providers’ well-being. This review identifies avenues for future research in this field including methodological advances by using quasi-experimental and prospective designs, representative samples, and adequate confounder control.

**Trial registration:**

Protocol registration number: PROSPERO 2016 CRD42016037220

## Introduction

Emergency department (ED) work systems are characterized by various psychosocial risk factors, e.g., high time pressure, varying workloads, and frequent exposure to potentially traumatic events [1, 2]. High rates of occupational stress and significant risks for burnout are reported by ED providers, e.g., by up to 26% of emergency nurses and over 35% of emergency physicians [3, 4]. A growing literature base emphasizes the key role of psychosocial work factors with regard to adverse health outcomes in ED providers [1–6]. Moreover, adverse psychosocial work factors and poor provider health mitigate optimal patient care practices, e.g., by increasing the likelihood of medical errors and near misses, or patient dissatisfaction [7, 8]. So far, no systematic review aimed to quantify this growing research base to determine present methodological study quality in this field, and to inform respective interventions to promote ED physicians’ and nurses’ well-being in this highly demanding care environment.

According to work system theory, each work system encompasses elements of the physical environment, tasks, tools and technologies, organization, and employee factors [9]. All elements interact and produce physical, psychological, and cognitive stress loads on employees which in turn impact individual outcomes such as health, well-being, and work performance [10]. Persistent exposure to extensive job demands or imbalance between positive and negative work factors lead to psychological distress while well-designed work systems promote positive provider outcomes [9, 11, 12].

ED settings are clinical environments with unique characteristics compared to other hospital units. Available reviews on ED work stress only applied narrative aggregation and, therefore, lack quantitative synthesis of the variety of psychosocial work factors and associated provider outcomes [1–6]. In addition to current qualitative summaries and in order to develop effective prevention measures, we need to systematically gather and pool available information as well as establish systematic evidence to develop a reliable estimate of the influence of psychosocial work factors for ED providers’ well-being.

Thus, the main goals of this systematic review are (1) to identify and categorize psychosocial ED work factors associated with the mental well-being of ED providers, (2) to systematically categorize these relationships according to their quantity as well as strength, and (3) to derive recommendations for future research and prevention practice.

## Methods

A review protocol was registered and is available on PROSPERO, registration number: CRD42016037220. We followed the guidelines on Preferred Reporting Items for Systematic Reviews and Meta-Analyses (PRISMA) (S1 Table) [13]. Searches were run in October 2016 and updated in December 2017.

### Search strategy and study selection

We conducted a comprehensive literature search in eight databases: PubMed, MEDLINE, PsycINFO, Academic Search Complete, Business Source Complete, Embase, Scopus, and Web of Science core collection. Keywords were used in a multi-field search describing the study population, psychosocial work factors, and ED providers’ mental well-being (S2 Table).

All identified records were screened in consecutive steps (S3 Table). After removing duplicates, both authors independently screened all titles and abstracts of retrieved records based on inclusion and exclusion criteria described in Table 1:

**Table 1:** Study inclusion and exclusion criteria.

Criterion	Inclusion	Exclusion
Study design	• Quantitative observational studies • Published in peer-reviewed journals • Published between 1996 and December 2017 • Published in English or German	• Other study types, including case reports, conference abstracts and proceedings, qualitative studies, and experimental studies
Population	• ED nurses and physicians • Other providers regularly employed in EDs (i.e., technicians, administrative staff)	• Emergency medical services personnel working in pre-hospital settings • Consultants from hospital units outside the ED
Psychosocial work factors	• Psychosocial work factors and job characteristics derived from ED providers’ self-reports or expert observations	• Extraordinary work circumstances in ED care, e.g., service during natural disasters • Contextual variables of the work environment, e.g., patient numbers, shift work schedule • Person-specific variables, e.g., individual working hours, type of contract
ED providers’ mental well-being	• All mental well-being outcomes derived from individual ED providers’ self-reports or expert evaluations	• Global organizational-level outcomes, e.g., overall staff turnover rates or sick leave rates
Analytic methodology	• Bi- or multivariate associations between independent measurements of psychosocial work factors and well-being outcomes, i.e., associations between discrete variables	• Other descriptive approaches, e.g., frequency of variables which combine determinant and outcomes

ED: emergency department.

Initial agreement between authors in study selection from abstract screening was 90.8% for 1473 records. Consensus over final inclusion of studies was reached through discussion. Full texts of included records were retrieved. Authors of unavailable articles were contacted. The first author (AS) reviewed all available full texts. N = 100 full texts were further independently assessed for eligibility by the second author (MW). Disagreement over inclusion was resolved through discussion until consensus was achieved. Further eligible studies were searched in references of full texts and in previous reviews on similar topics [1–6]. The first author (AS) extracted data from original studies according to a predefined scheme including information on (1) study title, authors, year of publication; (2) ED setting country, ED type and specialty, hospital type, number of annual visits); (3) study design and data collection methods; (4) sample characteristics (ED providers, population size, sample size, response rate, age, gender); (4) determinant and outcome variables (assessment instruments, information on validity and reliability of measures); (5) statistics (statistical methods, power calculation, reported associations, contextual variables); and (6) other relevant information (ethics approval, informed consent, compensation) (S4 Table).

Both authors independently assessed all included studies for methodological quality and risk of bias with the Quality Assessment Tool for Quantitative Studies from the Effective Public Health Practice Project (EPHPP) [14]. EPHPP lists several quality criteria and is suitable for systematic reviews combining original research with different study designs [15]. Inconsistencies in ratings were resolved through discussion until consensus was reached. Studies were not excluded from further analysis and quantitative synthesis on the basis of quality ratings.

### Analysis and synthesis

One author (AS) extracted and classified all univariate and multivariate associations into weak, moderate, or strong according to conventional cut-off criteria for correlational effect sizes [16], group differences, and risk estimates [17] (S5 Table). Effect sizes were differentiated into uncontrolled (univariate) and controlled (multivariate) associations, because results from multivariate techniques allow for the assessment of one particular determinant variable while simultaneously taking into account the effects of other potentially relevant determinant factors [18]. Multivariate associations are preferred because they are partly controlled for confounding influences.

Both authors assigned psychosocial work factors to a multi-level taxonomy drawing on the work system model [9]: (a) patients and task-related work factors, e.g., job control, work overload; (b) organizational factors, e.g., personnel resources, rewards; (c) social factors, e.g., support from supervisors or colleagues, interpersonal conflict; and (d) other factors which could not be assigned to (a)–(c), such as general job demands (S5 Table).

ED providers’ mental well-being outcomes were classified into (i) positive well-being outcomes, e.g., job satisfaction, work engagement; or (ii) affective symptoms and negative psychological functioning, e.g., emotional exhaustion, post-traumatic stress reactions; or (iii) cognitive-behavioural outcomes, e.g., turnover intention, commitment, and role behaviours; or (iv) health complaints, e.g., somatic symptoms, physical complaints (S5 Table).

In this study, we applied harvest plots to summarize the number and strength of associations between categories of psychosocial work factors and well-being in ED providers (S6 Table). Previous reviews omitted a systematic aggregation of the magnitude of observed associations between psychosocial work factors and ED provider well-being. Yet, in order to identify key risk factors in the ED work environment as well as to develop effective interventions in this field, the distribution of identified associations needs to be collated and illustrated. Thus, in addition to a systematic description of included studies, we applied harvest plots as an innovative approach to graphically pool information and to synthesize quantitative results. Harvest plots are an informative and comprehensive mode of presenting results of systematic reviews and are recommended particularly in case of non-applicability of meta-analysis, i.e., due to substantial heterogeneity of methodological characteristics, populations, study variables, and outcomes [19, 20]. Similar to forest plots, harvest plots display the distribution of evidence for a specific set of hypotheses through a customized und user-friendly structure. Additionally, analyses for ED physicians and nurses were compiled, i.e., harvest plots for each ED profession (S6 Table).

## Results

**Fig 1:**
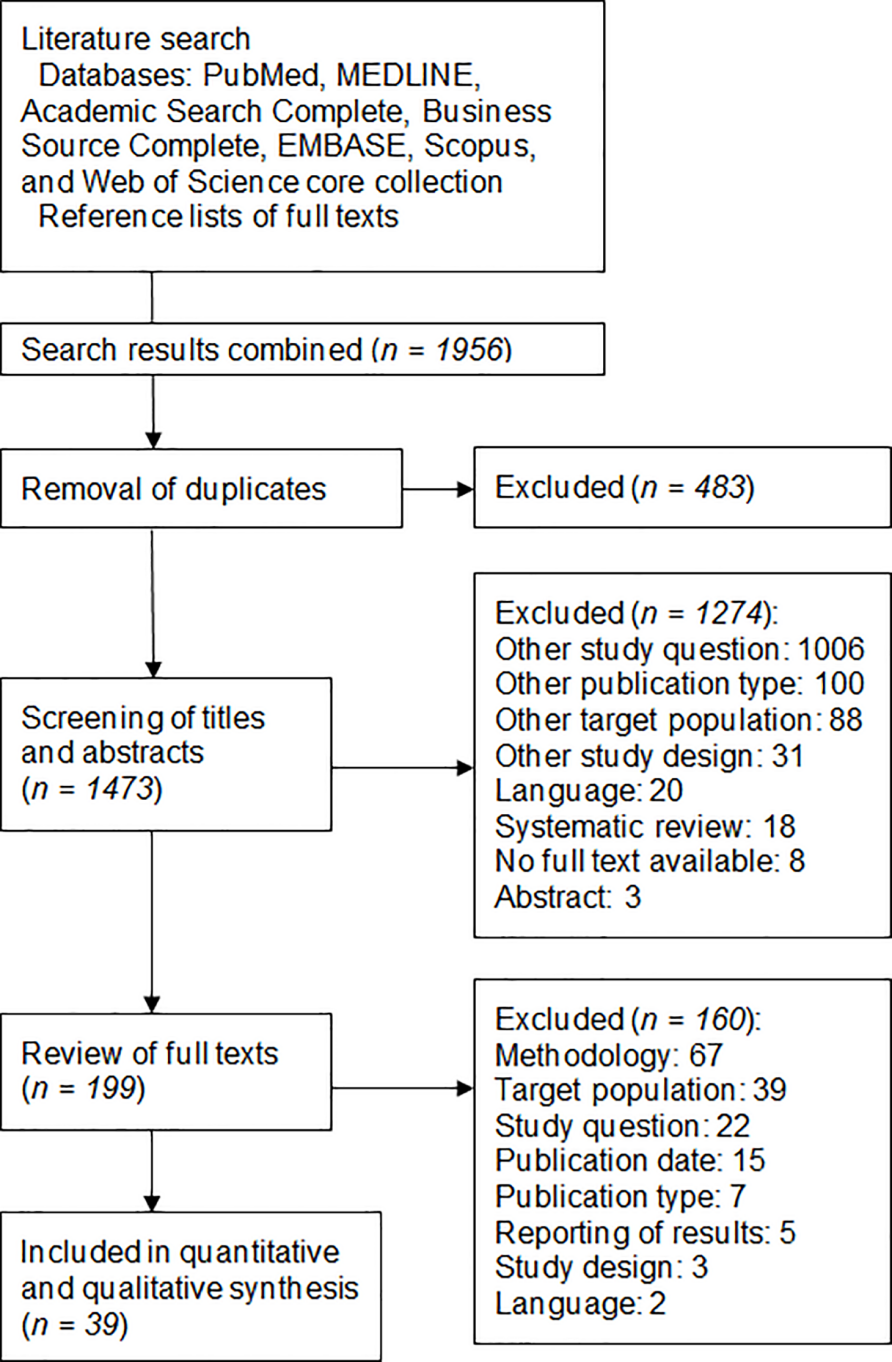
Flow diagram of study selection.

Thirty-nine studies were eligible for inclusion after the screening and selection process (flow chart in Fig 1).

Table 2 describes key characteristics of 39 included studies. Thirty-seven studies used a cross-sectional design, whereas two applied a prospective design [21, 22]. Data collection methods were paper or mail questionnaires (33 studies), online surveys [23–25], combined surveys [26], or structured interviews [27, 28]. Thirteen studies were conducted in European [21, 24, 26, 29–38] and twelve in (primarily North)-American settings [22, 23, 27, 39–47]. Another twelve studies originated in Asia [25, 28, 48–57] and one each in Africa [58] and in Australia [59]. Four studies used a single-centre approach [35, 37, 41, 59]. Multi-centre designs varied in eight studies with 2 to 10 EDs [28, 31, 34, 36, 39, 51, 57, 58], nine studies with 11 to 20 EDs [21, 29, 30, 38, 43, 50, 53, 55, 56], and three studies with 112 to 168 EDs [27, 49, 52]. Fifteen studies did not provide information on the number of surveyed EDs [22–26, 32, 33, 40, 42, 44–48, 54].

**Table 2:** Key characteristics of included studies on psychosocial work factors and mental well-being in ED providers.

First author, publication year, country, and citation	Participants (P); Response rate (R)	Key study variables and measures	
		Determinant variables: Psychosocial work factors (survey instruments)	Outcome variables: Mental well-being (survey instruments)
1 Adriaenssens, 2015, Belgium [21]	P: 170 nurses, 15 EDs; R: T1: 82.5%; T2: 83.3%	(a) Job demands, job control; (b) Work agreements, material resources, personnel resources, rewards; (c) Social support, social harassment (all LQWQ-N)	(i) Job satisfaction (LQWQ-N), work engagement (UWES); (ii) Emotional exhaustion (MBI), psychosomatic distress (BSI); (iii) Turnover intention (LQWQ-N)
2 Adriaenssens, 2012, Belgium [29]	P: 248 nurses, 15 EDs; R: 80.5%	(a) Frequency of exposure to traumatic events (self); (c) Social support from supervisor, social support from colleagues (both LQWQ-N)	(ii) Posttraumatic stress reactions (IES), psychological distress (BSI); (iv) Fatigue (CIS-20R), somatic complaints (BSI), sleep problems (self)
3 Adriaenssens, 2011, Belgium [30]	P: 254 nurses, 15 EDs; P: 82.5%	(a) Work/time demands, decision authority, skill discretion, physical demands; (b) Personnel resources, work procedures, material resources, rewards; (c) Social support from supervisor, social support from colleagues (all LQWQ-N)	(i) Job satisfaction (LQWQ-N), work engagement (UWES); (ii) Psychosomatic distress (BSI); (iii) Turnover intention (LQWQ-N); (iv) Fatigue (CIS-20R)
4 Ben-Itzhak, 2015, Israel [53]	P: 70 physicians, 16 EDs; R: 35%	(a) Meaningful job; (b) Work/life balance; (c) Social support (all self)	(ii) Burnout (MBI)
5 Blando, 2013, USA [27]	P: 314 nurses, 168 EDs; R: n.d.	(a) Assaults, verbal abuse; (b) Violence-based safety training, security equipment, security guards, security response time, importance of security to management, reports about violence, information about violent events; (c) Security and ED staff working together (all self)	(i) Feelings of safety (self)
6 Bruyneel, 2016, Belgium [38]	P: 294 nurses, 11 EDs; R: 69.7%	(a) Work/time demands, decision authority, skill discretion, physical demands (all LQWQ-N); (b) Nurse foundations for quality of care, nurse participation in hospital affairs, nurse staffing, career development and opportunities, nurse management and leadership (all PES-NWI); (c) Collegial nurse/physician relations (PES-NWI), social support from supervisor and colleagues (LQWQ-N)	(i) Job satisfaction (LQWQ-N); (ii) Emotional exhaustion (MBI-HSS); (iii) Turnover intention (LQWQ-N)
7 Chen, 2017, Taiwan [54]	P: 398 physicians; R: 39%	(a) Workload; (b) Emergency safety, salary and benefit; (d) Supporting environment (all self)	(i) Well-being/ happiness; (iii) Turnover intention (all self)
8 Clem, 2008, USA [23]	P: 1380 female physicians; R: 56%	(b) Compensation, career advancement, recognition, schedule flexibility, equal advancement opportunities and equal compensation for men/women; (c) Interactions with nurses/ non-physicians, appreciation by supervisor, relationship with colleagues (all self)	(i) Career satisfaction (self)
9 Converso, 2015, Italy [31]	P: 95 nurses, 2 EDs; R: n.d.	(a) Job autonomy, psychological demands (both JCQ), gratitude (PGRate) and support from patients (CIS)	(i) Personal accomplishment; (ii) Emotional exhaustion, depersonalization (all MBI-HSS)
10 Crilly, 2017, Australia [59]	P: 34 nurses; R: T1: 33%	(a) Self-realization, workload; (c) Conflict (WES-10)	(ii) Nervousness (WES-10)
11 Cydulka, 2008, USA [22]	P: T1: 945, T2: 823, T3: 771 physicians; R: T1: 94%, T2: 82%, T3: 76%	(a) Energy needed for work, exciting work, control over working conditions, knowing enough, level of patient acuity; (b) Time for personal life, hospital administration, length of shifts, subspecialty support, compensation, job security, personal reward, night shifts, opportunity to attend conferences; (c) Relationship with colleagues (all self)	(i) Career satisfaction; (ii) Burnout (all self)
12 Escriba-Aguir, 2006, Spain [32]	P: 630 physicians and nurses; R: 67.6%	(a) Psychological-emotional demands, job control, physical workload; (c) Social support from supervisor, social support from colleagues (all JCQ)	(i) Personal accomplishment; (ii) Emotional exhaustion, depersonalization (all MBI)
13 Escriba-Aguir, 2007, Spain [33]	P: 630 physicians and nurses; R: 67.6%	(a) Psychological demands, job control, physical workload; (c) Social support from supervisor, social support from colleagues (all JCQ)	(i) Vitality (SF-36); (ii) Emotional exhaustion (MBI), mental health (SF-36)
14 Estryn-Behar, 2011, France [24]	P: 538 physicians; R: n.d.	(a) Influence at work (DC), quantitative demands (COPSOQ and self), violence from patients/relatives (self); (b) Work/family conflict (WFC); (c) Interpersonal relationships within team, relationships with administration, harassment by superiors, support from colleagues (all self)	(ii) Burnout (CBI); (iii) Intention to leave (self)
15 Garcia-Izquierdo, 2012, Spain [34]	P: 191 nurses, 3 EDs; R: 73%	(a) Excessive workload, death and suffering; (b) Lack of resources; (c) (Interpersonal) conflicts, lack of social and emotional support (all NSS)	(i) Professional efficacy; (ii) Emotional exhaustion, cynicism (all MBI)
16 Gates, Ross, 2006, USA [39]	P: 242 workers, 5 EDs; R: n.d.	(a) Verbal and sexual harassment, threats, assaults (all self)	(i) Feelings of safety (self)
17 Hamdan, 2017, Palestine [56]	P: 444 physicians, nurses, admission personnel; R: 74.5%	(a) Exposure to physical violence, exposure to non-physical violence (self)	(i) Personal accomplishment; (ii) Emotional exhaustion, depersonalization (all MBI)
18 Hamdan, 2015, Palestine [55]	P: 444 physicians, nurses, admission personnel; R: 74.5%	(a) Exposure to physical violence, exposure to non-physical violence (self)	(iii) Intention to quit (self)
19 Hsieh, 2016, Taiwan [28]	P: 159 nurses, 2 EDs; R: 88.3%	(c) Peer support (SSS)	(i) Resilience (RS); (ii) Depression (CES-D)
20 Hunsaker, 2015, USA [40]	P: 284 nurses; R: 28%	(c) Support from manager (self)	(i) Compassion satisfaction; (ii) Burnout, compassion fatigue (all ProQOL 5)
21 Jalili, 2013, Iran [48]	P: 165 physicians; R: 88%	(a) Text needed to be read, patients’ economic problems, patient overload, skills, violence, care of old/terminally ill patients; (b) Shortage of equipment, physical environment, problems with other services, economic problems/future of EM career, imbalance of professional/private life, educational issues, image of EM in media, consultant unavailability, new information and technologies; (c) Lack of support and encouragement, communication with colleagues (all self)	(i) Personal accomplishment; (ii) Emotional exhaustion, depersonalization (all MBI)
22 Kogien, 2014, Brazil [41]	P: 189 nurses and technicians, 1 ED; R: n.d.	(a) Intellectual discernment; (c) Social support; (d) Work demands (all JSS)	(iv) Physical domain of quality of life (WHOQOL-BREF)
23 Lin, 2011, Taiwan [49]	P: 385 nurses and physicians, 112 EDs; R: n.d.	(b) Task- and employee-oriented leadership (self)	(i) Satisfaction; (iii) Unit performance (both self)
24 Lin, 2012, Taiwan [52]	P: 442 physicians and nurses, 119 EDs; R: n.d.	(b) Clan culture, adhocracy culture, market culture, hierarchy culture (all OCAI)	(iii) Intent to leave (self)
25 O'Mahony, 2011, Ireland [35]	P: 64 nurses, 1 ED; R: 74%	(a) Time to discuss patient care; (b) Quality assurance program, administration consults, non-punitive management, high standards by administration, administration listens/responds; (c) Nurse/physician collaboration, teamwork (all NWI-PES)	(ii) Emotional exhaustion, depersonalization (all MBI)
26 Revicki, 1997, USA [42]	P: 484 physicians; R: 50% to 55%	(a) Role ambiguity (self, MOAQ); (c) Peer (self) and work-group support (self, MOAQ)	(i) Work satisfaction (self, MOAQ); (ii) Work stress (WRSI), depression (CES-D)
27 Rios-Risquez, 2016, Spain [36]	P: 148 nurses, 2 EDs; R: 73%	(d) Frequency of stress (NSS)	(i) Personal effectiveness; (ii) Emotional exhaustion, cynicism (all MBI-GS)
28 Sawatzky, 2012, Canada [43]	P: 261 nurses, 12 EDs; R: 35%	(a) Competence, professional practice; (c) Work overtime, staffing resources, nursing management; (c) Collaboration with physicians (all PNWE)	(i) Job satisfaction (self), engagement (ECQ), compassion satisfaction (ProQOL); (ii) Compassion fatigue, burnout (both ProQOL); (iii) Intention to leave (nursing) (Price&Mueller)
29 Somville, 2016, Belgium [26]	P: 181 physicians; R: 43.9%	(a) Physical hazards, violence (both Dorevitch et al.), traumatic events (self); (b) Supervisor and colleagues support (both LQWQ-MD)	(i) Job satisfaction (LQWQ-MD); (ii) Posttraumatic stress reactions (IES), psychological distress (BSI); (iv) Somatization (PHQ 15), fatigue (CIS-20R)
30 Sorour, 2012, Egypt [58]	P: 58 nurses, 2 EDs; R: n.d.	(d) Job demands (JCQ)	(ii) Burnout (MBI)
31 Taylor, 2004, USA [44]	P: 323 physicians; R: 63.5%	(a) Control of activity mix; (b) Control of hours worked (both self)	(i) Work (self) and life satisfaction (SLS); (ii) Work stress (PSS), depression (ZDS), anxiety (ZAS); (iv) Physical symptoms (PSC)
32 Toker, 2015, Turkey [25]	P: 167 physicians; R: 40.7%	(a) Appreciation by patients/ relatives, exposure to violence; (b) Presence of consultant; (c) Compliance with personnel, appreciation by supervisor and co-workers (all self)	(i) Personal accomplishment; (ii) Emotional exhaustion, depersonalization (all MBI)
33 Trautmann, 2015, USA [45]	P: 246 nurse practitioners; R: 31%	(d) Practice independence (DPBS)	(iii) Intention to leave (MDS-R)
34 Weigl, 2016, Germany [37]	P: 53 staff members, 1 ED; R: 61.6%	(a) Autonomy, time pressure, patient-related stressors; (b) Staffing; (c) Supervisor support (WDQ)	(ii) Emotional exhaustion (MBI), irritation (Irri)
35 Williams, 2007, Canada [46]	P: 428 physicians; R: 29.8%	(b) Culture (bureaucratic/ human resources/ entrepreneurial/ rational) (all self)	(iii) Patient commitment, extra-role behaviour (all self)
36 Wilson, 2017, India [57]	Pt: 105 physicians and nurses; R: n.d.	(a) Affected by high mortality, increased load of patients, infection risk; (c) More criticism, departmental activities for staff bonding (all self)	(i) Personal accomplishment; (ii) Emotional exhaustion, depersonalization (all MBI)
37 Wu, 2012, China [50]	P: 510 female nurses, 16 EDs; R: 77.9%	(a) Role overload, role insufficiency, role ambiguity, role boundary, responsibility (all self)	(ii) Occupational stress (PSQ)
38 Young-Ritchie, 2009, Canada [47]	P: 206 nurses; R: 73%	(b) Emotionally intelligent leadership (ECI 2.0), structural empowerment (CWEQ-II)	(iii) Affective commitment (T-C MEC)
39 Zahid, 1999, Kuwait [51]	P: 101 physicians; R: 68.7%	(a) Violence (self)	(ii) Depression, reliving experiences, fearfulness; (iii) Time off; (iv) Sleeplessness (all self)

n.d.: not described; self: self-developed questions; Categorization for psychosocial work factors: (a) patients and tasks, (b) organizational factors, (c) social factors, (d) other factors; Categorization for mental well-being: (i) positive well-being, (ii) affective symptoms and negative psychological functioning, (iii) cognitive-behavioural outcomes, (iv) health complaints; LQWQ-(N or MD): Leiden Quality of Work Questionnaire (for Nurses or for Medical Doctors), UWES: Utrecht Work Engagement Scale, MBI-(HSS or GS): Maslach Burnout Inventory (Human Services Survey or General Survey), BSI: Brief Symptom Inventory, IES: Impact of Event scale, CIS-20R: Checklist Individual Strength, CISS: Customer-initiated Support Scale, JCQ: Job Content Questionnaire, SF-36: SF-36 Health Survey, WFC: Work-family Conflict Scale, COPSOQ: Copenhagen Psychosocial Questionnaire, DCQ: Demand-Control Questionnaire, CBI: Copenhagen Burnout Inventory, NSS: Nursing Stress Scale, SSS: Social Support Scale, RS: Resilience Scale, CES-D: Centre for Epidemiologic Studies Depression, ProQOL 5: Professional Quality of Life Version 5, JSS: Job Stress Scale, WHOQOL-BREF: World Health Organization Quality of Life Short Version, NWIPES: Nursing Work Index Practice Environment Scale, WRSI: Work-Related Strain Inventory, PNWE: Perceived Nurse Working Environment, ECQ: Engagement Composite Questionnaire, PHQ 15: Prime MD Patient Health Questionnaire, ZDA: Zung Depression Scale, ZAS: Zung Anxiety Scale, PSC: Physical Symptoms Checklist, PSS: Perceived Stress Scale, SLS: Satisfaction with Life Scale, JSS: Job Satisfaction Scale, DPBS: Dempster Practice Behavior Scale, MDS-R: Moral Distress Scale-Revised, PSQ: Occupational Stress Inventory, PCL-C: PTSD CheckList–Civilian Version, ECI 2.0: Emotional Competency Inventory, CWEQ-II: Conditions of Work Effectiveness Questionnaire–II, T-CMECS: Three-Component Model Employee Commitment Survey, OCAI: Organizational Culture Assessment Instrument, WDQ–Work-Demand Questionnaire, Irri: Irritation Scale, PES-NWI: Practice Environment Scale of the Nursing Work Index, WES-10: Working Environment Score (10-item version).

### Study population

Concerning sampled ED professions, 18 studies explicitly focused on nurses [21, 27–31, 34–36, 38, 40, 41, 43, 45, 47, 50, 58, 59], 12 on physicians [22–26, 42, 44, 46, 48, 51, 53, 54], while three interrogated multi-professional samples [32, 33, 57]. Four studies further involved non-clinical ED professions including administrative and support staff [37, 39, 55, 56]. Two studies used EDs as units of analyses [49, 52]. Ten studies likely used similar samples for different study questions [21, 29, 30, 32, 33, 38, 49, 52, 55, 56]

Median study population size was 465 for physician samples, 378 for nurse samples, and 419 for multi-professional samples. Nine studies did not describe population size characteristics [24, 27, 31, 41, 42, 49, 52, 57, 58]. Median final sample size for physician samples was 225, 242 for nurse samples, and 225 for multi-professional samples. In 11 out of 12 studies on physician samples with specifications of gender, the majority of participants were male. One study included solely female emergency physicians [23]. In contrast, in studies which specified gender in nurse samples, 13 out of 14 included more than 50% female participants; only one study reported a slight surplus of male nurses [38].

### Quality ratings

All included studies were evaluated with the EPHPP tool for methodological quality and risk of bias [14]. None of the 39 included studies achieved a strong overall appraisal (Table 3). Eleven studies attained moderate ratings [21, 24, 30, 32–34, 38, 41, 48, 49, 58]. The remaining twenty-eight studies suggested a heightened risk of bias with overall weak ratings. Concerning individual quality categories, 35 out of 39 included studies received weak or moderate ratings on selection bias, indicating insufficient study sample representativeness or low response rates. Considering control for potential confounders in study design or analyses, 29 out of 39 studies were evaluated as weak or moderate, indicating limited control for potential confounders. However, 25 out of 39 studies obtained a strong rating for data collection due to the application of valid and reliable measurement methods. None of the included studies achieved a strong rating in the remaining three categories, which was mostly due to their cross-sectional design, i.e., concerning study design, withdrawals, and inability to blind outcome assessors and study participants.

**Table 3:** Quality rating for included studies according to the Quality Assessment Tool for Quantitative Studies (EPHPP).

	Included studies (first author, year)																																						
	1	2	3	4	5	6	7	8	9	10	11	12	13	14	15	16	17	18	19	20	21	22	23	24	25	26	27	28	29	30	31	32	33	34	35	36	37	38	39
Selection bias	+	+	+	-	0	0	-	-	0	-	0	0	0	0	0	0	0	0	0	-	+	0	0	0	0	-	0	-	-	0	0	-	-	0	-	-	0	0	0
Study design	0	-	-	-	-	-	-	-	-	-	0	-	-	-	-	-	-	-	-	-	-	-	-	-	-	-	-	-	-	-	-	-	-	-	-	-	-	-	-
Confounders	-	-	+	-	-	0	-	0	-	-	+	+	+	+	0	-	-	-	-	-	+	0	+	+	-	-	-	+	-	0	-	-	+	-	-	-	-	-	-
Blinding	0	0	0	0	0	0	0	0	0	0	0	0	0	0	0	0	0	0	0	0	0	0	0	0	0	0	0	0	0	0	0	0	0	0	0	0	0	0	0
Data collection	+	+	+	-	0	+	-	-	+	+	-	+	+	+	+	-	+	+	+	+	0	0	+	-	+	+	+	+	0	+	-	+	+	+	-	0	+	+	-
Withdrawals	0	0	0	0	0	0	0	0	0	-	-	0	0	0	0	0	0	0	0	0	0	0	0	0	0	0	0	0	0	0	0	0	0	0	0	0	0	0	0
**Global rating**	0	-	0	-	-	0	-	-	-	-	-	0	0	0	0	-	-	-	-	-	0	0	0	-	-	-	-	-	-	0	-	-	-	-	-	-	-	-	-

EPHPP: Effective Public Health Practice Project; + strong rating; 0 moderate rating;—weak rating.

### Associations between psychosocial work factors and well-being

First, univariate associations of eligible studies were extracted. Overall, 367 univariate associations between psychosocial work factors and provider well-being were identified, whereof 261 associations (71.1%) were reported as statistically significant, indicated with a probability level of p<0.05. Second, 370 specific multivariate associations from included studies were extracted, whereof 149 associations (40.3%) were significant.

**Fig 2:**
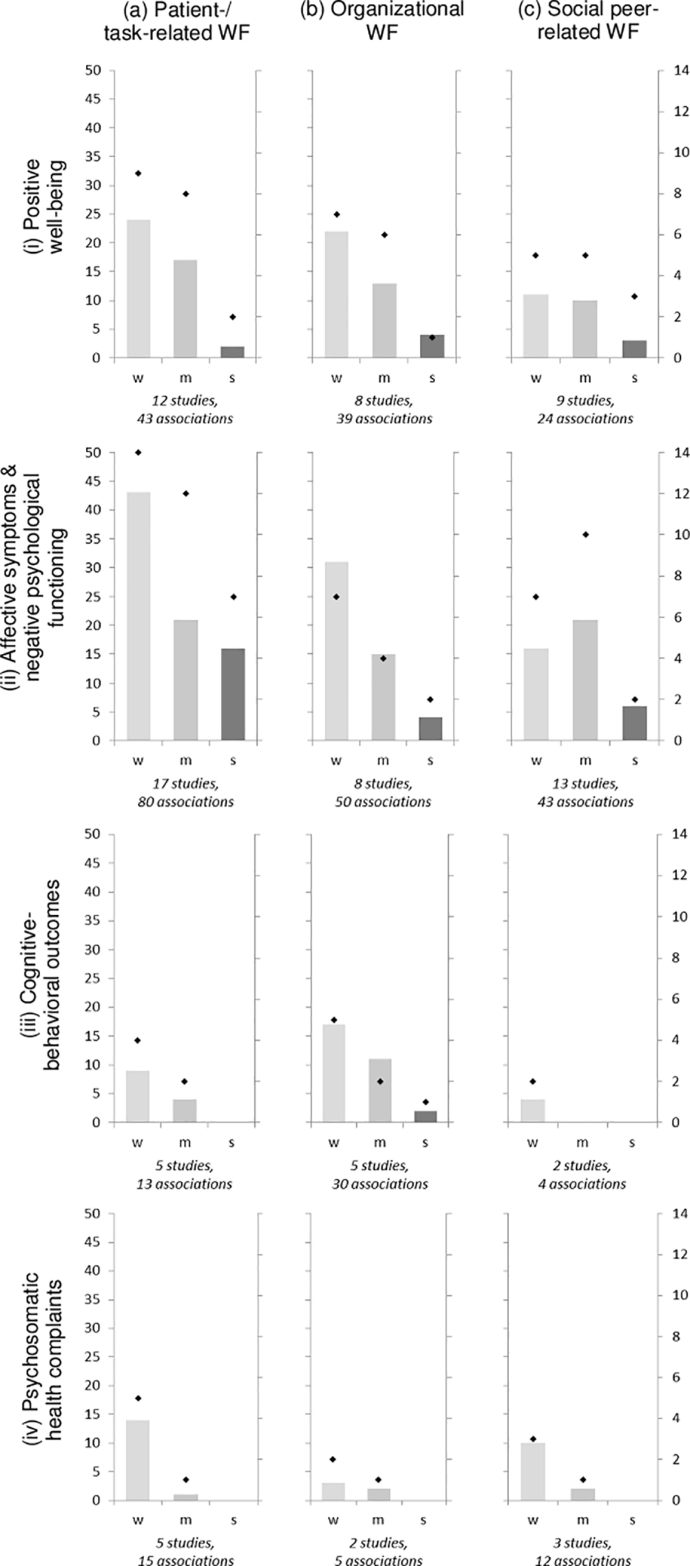
Harvest plot of univariate associations between psychosocial work factors (WF) and ED providers’ mental well-being. Left axis (bars) denominates frequency of univariate associations; right axis (diamonds) denominates number of original studies describing these relationships; w: weak, m: moderate, s: strong; Text in italics denominates total number of original studies and total number of univariate associations analysing variables out of the respective categories for psychosocial work factors and mental well-being outcomes.

**Fig 3:**
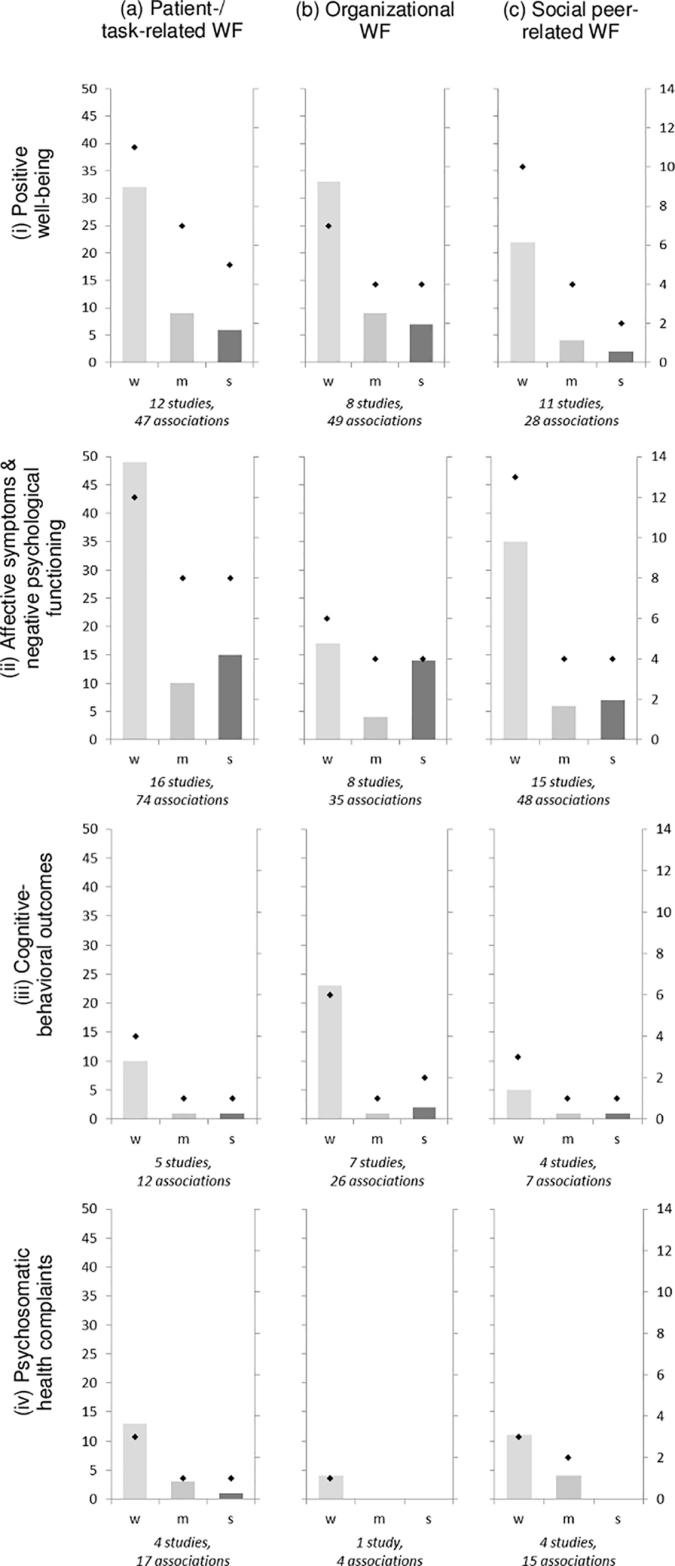
Harvest plot of multivariate associations between psychosocial work factors (WF) and ED providers’ mental well-being. Left axis (bars) denominates frequency of multivariate associations; right axis (diamonds) denominates number of original studies describing these relationships. W: weak, m: moderate, s: strong; Text in italics denominates total number of original studies and total number of multivariate associations analysing variables out of the respective categories for psychosocial work factors and mental well-being outcomes.

The range and heterogeneity of different constructs and measurement instruments across studies allowed no valid base for meta-analysis. Therefore, two harvest plots for results of univariate and multivariate associations were compiled (Figs 2 and 3). Harvest plots depict the total amount and strength of identified associations between categorized psychosocial work factors and four categories of well-being outcomes, respectively. Due to varying measurement approaches and operationalization of study variables in included studies, harvest plots do not differentiate between positive or negative directions of association. Further, since sample size affects power of statistical tests [60] and biases may influence p values [18], all associations from original studies were included into harvest plots irrespective of their reported level of significance. Category (d) general work factors was omitted from further graphical analyses due to its low allocation status (n = 17 associations). Separate analyses for ED nurse and physicians samples are presented in additional harvest plots (S1–S4 Figs).

#### Number of identified associations

Across all included studies, ED providers’ affective symptoms and negative psychological functioning (n = 26 studies) as well as positive well-being outcomes (n = 21) were most commonly investigated. In contrast, cognitive-behavioural outcomes (n = 12 studies) and psychosomatic health complaints (n = 6) were less often surveyed. Patient- and task-related factors (n = 29 studies) were most frequently analysed in relation to mental well-being, followed by social (n = 23), and organizational factors (n = 19).

#### Strength of identified associations

The following patterns were observed for all extracted univariate associations (Fig 2): for (i) *positive well-being outcomes*, the highest percentage of strong and moderate associations was found for social work factors (12.5% and 41.7%, respectively). In nursing professionals, however, organizational work factors showed most strong associations (see S1 Fig; 16.7%). For (ii) *affective symptoms and negative psychological functioning*, patient- and task-related work factors had the largest amount of strong associations (see Fig 2; 20%) and social work factors the largest amount of moderate associations (48.8%). In physician samples, social factors held the largest amount of strong associations (see S3 Fig; 25%). For (iii) *cognitive-behavioural outcomes*, organizational work factors had the largest amount of strong and moderate associations (see Fig 2; 6.7% and 36.7%, respectively). However, for physician samples, no strong and moderate associations were observed (see S3 Fig). For (iv) *health complaints*, none of the included work factors were associated strongly (see Fig 2). Organizational work factors showed the largest amount of moderate associations (40%). In physician samples, social work factors were most often associated with moderate strength (see S3 Fig; 50%).

For multivariate associations slightly different patterns were observed (see Fig 3): For (i) *positive well-being outcomes*, the largest amount of strong and moderate associations was found for organizational work factors (14.3% and 18.4%, respectively), comparable to patient- and task-related work factors (12.8% and 19.1%, respectively). For (ii) *affective symptoms and negative psychological functioning*, organizational work factors held the largest amount of strong associations (40.0%) and patient- and task-related (13.5%) as well as social work factors (12.5%) the largest shares of moderate associations. For (iii) *cognitive-behavioural outcomes*, social factors had the largest amount of strong and moderate associations (both 14.3%). However, in nurse samples, organizational (7.1%) and patient-/task-related work factors (9.1%) had the largest share of strong and moderate associations, respectively (S2 Fig). Finally, for (iv) *health complaints*, patient- and task-related work factors were most often associated strongly (see Fig 3; 5.9%), however, social work factors held the largest count of moderate associations (26.7%).

### Effects of specific psychosocial work factors on mental well-being

In a final step, we identified all statistically significant associations between psychosocial work factors and ED providers’ well-being outcomes (S7 Table). This procedure summarizes the most important findings stated in included studies and denominates specific psychosocial work factors as starting points for further analyses or interventions. The following associations deserve particular consideration:

#### Positive mental well-being outcomes

ED providers’ job satisfaction was most frequently examined, followed by work engagement, and personal accomplishment. Patient- or task-related factors, e.g., high job autonomy or job control and positive interactions with patients were associated with increased positive well-being [21, 22, 30–32, 38, 44]. In contrast, violence and harassment as well as work overload were detrimental to positive well-being [27, 39, 54, 57]. Organizational factors, e.g., schedule flexibility, participation opportunities, staffing, leadership quality, and adequate salary were positively associated with positive well-being [14, 22, 23, 38, 43, 44, 48, 49, 54]. Social support by colleagues or supervisors, and good teamwork also improved ED providers’ wellness [21, 23, 26, 28, 30, 33, 34, 38, 40, 42, 43].

#### Affective symptoms and negative psychological functioning

Burnout and its components were by far most frequently surveyed, followed by other affective symptoms such as depression, irritation, and psychological distress. PTSD and anxiety were less often examined. Patient- or task-related factors, e.g., workload, time pressure, violence, and traumatic events had adverse effects on affective symptoms and negative psychological functioning [22, 25, 26, 29, 30, 34, 35, 37, 38, 48, 51, 56, 57]. In contrast, job autonomy and positive interactions with patients were associated with less negative well-being [22, 25, 31, 32, 38, 44, 59]. Organizational factors, e.g., staffing problems, difficulties with administration, work-family conflict, unfair compensation or rewards contributed to increased negative affective symptoms [22, 24, 25, 34, 35, 38, 43, 48]. Again, favourable social factors such as good relationships with colleagues, teamwork, appreciation and support from supervisors were associated with fewer negative outcomes [21, 25, 28–30, 32, 33, 35, 37, 38, 40, 42].

#### Cognitive-behavioural outcomes

Turnover intentions were most frequently analysed [21, 24, 30, 38, 43, 52, 54, 55]. Other outcomes included patient commitment [46, 47] and extra-role behaviour [46]. Favourable psychosocial work factors for positive cognitive behavioural-outcomes such as less turnover intentions, more patient commitment, and extra-role behaviours were job control, influence at work, rewards, encouraging unit culture, leadership, and good relationships with supervisors.

#### Psychosomatic health complaints

This category included somatic complaints, sleep problems, or fatigue. Predominant predictors of impaired psychosomatic health on the patient- or task-related level were traumatic experiences, violence, and time pressure [26, 29, 30, 51]. Job control improved health complaints [41, 44]. Organizational factors such as rewards and work procedures contributed to fewer health complaints [30]. Beneficial social factors for this outcome category were social support from colleagues and supervisors [26, 29, 41].

## Discussion

To the best of our knowledge, this systematic review is the first that quantitatively synthesizes associations between psychosocial work factors and mental well-being in ED providers. A growing research base shows that well-designed ED work systems are fundamental to ED providers’ well-being and safe ED care [7, 9]. Yet, the field lacks a systematic appraisal of the current evidence as well as implications for future research and ED practice. We therefore collated the current research base on psychosocial risk factors and provider well-being outcomes and appraised its methodological quality. Our quality assessment indicated that none of the studies achieved a strong overall appraisal, with the majority evaluated as weak to moderate with considerable risk of bias. Methodological shortcomings of retrieved studies as well as potential methodological advances in the field will be discussed and proposed below. Nonetheless, taking these weak to moderate methodological foundation into account, the following contributions of this review need to be considered:

First, our review reveals a lack of research on psychosocial predictors of cognitive-behavioural outcomes and psychosomatic health complaints in ED providers, e.g., regarding turnover intentions or fatigue. The majority of included research investigated affective symptoms or positive well-being outcomes. Nonetheless, behavioural and health outcomes often result from a chronic exposure and a long-term impact of psychosocial work factors and occupational hazards [10, 61]. In comparison to frequently surveyed affective symptoms and positive well-being outcomes, ED providers’ turnover intentions and psychosomatic health complaints represent more distal well-being outcomes. These manifest particularly due to persistent exposure to adverse psychosocial work factors and failure to mitigate these stressors due to limited system or personal resources [38, 62]. Although ED work is often characterized by daily short-term peaks of work stress, prospective effects of chronic stressors and longstanding adverse work factors on ED professionals’ well-being need to be interrogated, i.e., in cohort studies. However, EDs are characterized by high staff turnover rates, partially due to high workloads and insufficient resources for providers [3] or rotation schedules during physician training, thus limiting possibilities for long-term follow-up in longitudinal research. This practical impediment remains a widely unaddressed issue of occupational health research in ED settings, which is also reflected in a dearth of longitudinal research identified in our systematic review [21, 22]. Moreover, future studies should test interactive and moderating relationships between psychosocial ED work factors, proximate mental well-being outcomes (i.e., stress, work strain), and, eventually, distal behavioural or health outcomes in ED providers [38].

Secondly, we found that the majority of relationships between psychosocial work factors and mental well-being were weak or moderate [16, 17]. However, strong associations were identified for the categories of social and organizational work factors and various well-being outcomes. Occupational health theories emphasize the importance of job resources as buffers in stressor-strain relationships. Thus maintaining good relationships with colleagues and supervisors enhances collaboration, strengthens individual resources, and alleviates the burden of adverse work conditions such as difficult interactions with patients or high workload [11, 61]. Therefore, our results highlight that key resources in EDs such as positive social relations, participation, and financial and non-tangible rewards buffer psychological demands and counteract adverse conditions of the ED work environment [9, 61].

### Limitations

According to PRISMA guidelines, review limitations need to be identified on two different levels, i.e., on study as well as the review level [13]:

Concerning the study-level, our review identifies alleys for further efforts to establish high quality studies with reinforced methodological rigour in this specific research field. Overall, the majority of included studies obtained only moderate to weak ratings in regard to methodological quality, with particular deficits regarding selection bias, study design, and control for confounders. The vast majority of studies applied cross-sectional designs that limit inferences concerning causality [18]. Accordingly, reverse or reciprocal causation between mental ill health and psychosocial work factors may occur over time and requires careful consideration [63]. Thus, different states of mental well-being could act as predictors for the appraisal of work conditions. Furthermore, the observed amount of statistically significant associations reported in included studies is striking and might indicate reporting or publication bias [18]. Future studies should also account for individual person-specific and other factors of the work system, e.g., those relating to contextual factors of the environment such as shift schedule or staffing. These factors were shown to influence providers’ mental health and well-being [9, 64]. Furthermore, external validity of our findings needs to be carefully considered since included studies originate from different hospital and national contexts as well as different health-care systems.

At review-level, further limitations apply. We restricted our search to quantitative studies that used separate measures of determinant and outcome variables. This approach facilitates reliable and valid conclusions on effect sizes of associations [65]. We acknowledge that previous reviews included studies with less robust methodological approaches [2–6]. Due to the substantial heterogeneity in populations and study methods as well as ambiguities and incomparability in measures, meta-analyses were not feasible. In this case of insufficient homogeneity to statistically combine data into meta-analyses, user-friendly and graphical summaries of evidence help decision makers and practitioners making sense of available evidence [20]. We thus applied harvest plots as an innovative and comprehensive approach that include the benefits of quantitative summaries without erroneously simplifying or falsely aggregating extracted relationships [19]. Our approach thus expands previous narrative reviews since it facilitates an improved understanding of the diverse and inconsistent research findings through comprehensive and graphical summaries of evidence. We pooled all included studies’ information and established different categories for psychosocial work factors and mental well-being. Future reviews in the field may draw upon our taxonomy to elicit a homogenous study and data base for statistical combination into first meta-analyses in the field. Nonetheless, potential misclassification of study variables due to missing or unspecified information in primary studies or plurivalent meanings of reported measures may have occurred. We categorized effect size magnitudes with conventional cut-off criteria that have been subject to scientific discourse [16, 17]. Finally, we applied a recommended and established tool to evaluate studies’ methodological quality [15]. However, during the rating process, some quality criteria of the EPHPP instrument were ambiguous with regard to cross-sectional and non-interventional designs, i.e., concerning withdrawals.

### Implications for future research and ED practice

This review systematically pooled information on the associations between psychosocial work factors and ED provider well-being and, additionally, appraised the methodological quality of research in this domain. Given the heterogeneity of retrieved studies, our approach is an intermediate but necessary step between existing narrative reviews and upcoming meta-analyses. Future reviews that seek to statistically quantify effects of psychosocial work factors and ED provider outcomes may draw upon our taxonomy for focus as well as to establish a homogenous study and data base. Our findings suggest further (a) to conduct controlled interventions and prospective studies that allow inferences concerning causation; (b) to recruit more representative study samples which enhance external validity; (c) to use standardized and validated questionnaires, objective measures, or expert evaluations; (d) and to apply adequate confounder control in study design or statistical analyses, and finally, (e) to consider effectiveness research on intervention approaches. There is a paucity of interventions that target psychosocial work factors in EDs [2]. Therefore, research on effective interventions to promote ED provider well-being is imperative and shall take account of our findings, particularly with regard key sources of occupational well-being in ED providers.

## Conclusions

This systematic review advances the current knowledge base on associations of psychosocial work factors and ED provider well-being with its quantitative focus, comprehensive aggregation of study findings, and rigorous evaluation of studies’ methodological quality. A multitude of different psychosocial risk factors characterizes the ED environment as a challenging and at times overtaxing work system. Especially social support and well-designed organizational systems were found to have a strong to moderate effect on ED providers’ well-being. System improvements in health care should be based on comprehensive evidence. However, the methodological foundations of our conclusions need to be considered carefully since methodological quality of included studies was low to moderate. On the one hand, our review informs future research endeavours in this field concerning robust study designs and assessment methods. On the other hand, our findings suggest starting points for work design interventions that address psychosocial work factors in order to promote providers’ well-being, retain ED providers in their jobs, and to improve clinical excellence.

## Supporting information

S1 Fig Harvest plot of univariate associations between psychosocial work factors (WF) and ED nurses’ mental well-being. Left axis (bars) denominates frequency of univariate associations; right axis (diamonds) denominates number of original studies describing these relationships; w: weak, m: moderate, s: strong; Text in italics denominates total number of original studies and total number of univariate associations analysing variables out of the respective categories for psychosocial work factors and mental well-being outcomes.
(TIF)

S2 Fig Harvest plot of multivariate associations between psychosocial work factors (WF) and ED nurses’ mental well-being. Left axis (bars) denominates frequency of multivariate associations; right axis (diamonds) denominates number of original studies describing these relationships. W: weak, m: moderate, s: strong; Text in italics denominates total number of original studies and total number of multivariate associations analysing variables out of the respective categories for psychosocial work factors and mental well-being outcomes.
(TIF)

S3 Fig Harvest plot of univariate associations between psychosocial work factors (WF) and ED physicians’ mental well-being. Left axis (bars) denominates frequency of univariate associations; right axis (diamonds) denominates number of original studies describing these relationships; w: weak, m: moderate, s: strong; Text in italics denominates total number of original studies and total number of univariate associations analysing variables out of the respective categories for psychosocial work factors and mental well-being outcomes.
(TIF)

S4 Fig Harvest plot of multivariate associations between psychosocial work factors (WF) and ED physicians’ mental well-being. Left axis (bars) denominates frequency of multivariate associations; right axis (diamonds) denominates number of original studies describing these relationships. W: weak, m: moderate, s: strong; Text in italics denominates total number of original studies and total number of multivariate associations analysing variables out of the respective categories for psychosocial work factors and mental well-being outcomes.
(TIF)

S1 Table PRISMA checklist. (DOC)

S2 Table Search strategy. (DOCX)

S3 Table Extracted records from literature search in databases and references. N: No; Y: Yes; X: applicable; N/A: not applicable; ft: full-text;?: uncertain.
(XLSX)

S4 Table Description of included studies. No.: number; ED: emergency department; N/A: not applicable / not available; T1: wave 1; T2: wave 2; SD: standard deviation; CA: Cronbach’s Alpha; r: correlation coefficient.
(XLSX)

S5 Table Extraction of statistical associations in original studies. No.: number; p-value: statistical probability; N: study sample size; T1: wave 1; T2: wave 2; ED: emergency department; UV: univariate association; MV: multivariate association; n.s.: not significant.
(XLSX)

S6 Table Numerical description of extracted associations for harvest plots. Number before brackets: number of extracted associations; number within brackets: number of studies describing these associations.
(XLSX)

S7 Table Categorized associations between psychosocial work factors and mental well-being outcomes. First author and year of publication in *italics*; Δ: delta/difference; T2: wave 2; β: standardized regression coefficient/beta; 95%CI: 95% confidence interval; OR: odds ratio; SPC: standardized path coefficient; r: correlation coefficient; SD: standard deviation.
(DOCX)
