# Supplementary figures and images for: Associations between psychosocial work factors and provider mental well-being in emergency departments: A systematic review
Source: PLoS One. 2018 Jun 4;13(6):e0197375. doi: 10.1371/journal.pone.0197375 (PMC5986127; doi:10.1371/journal.pone.0197375)

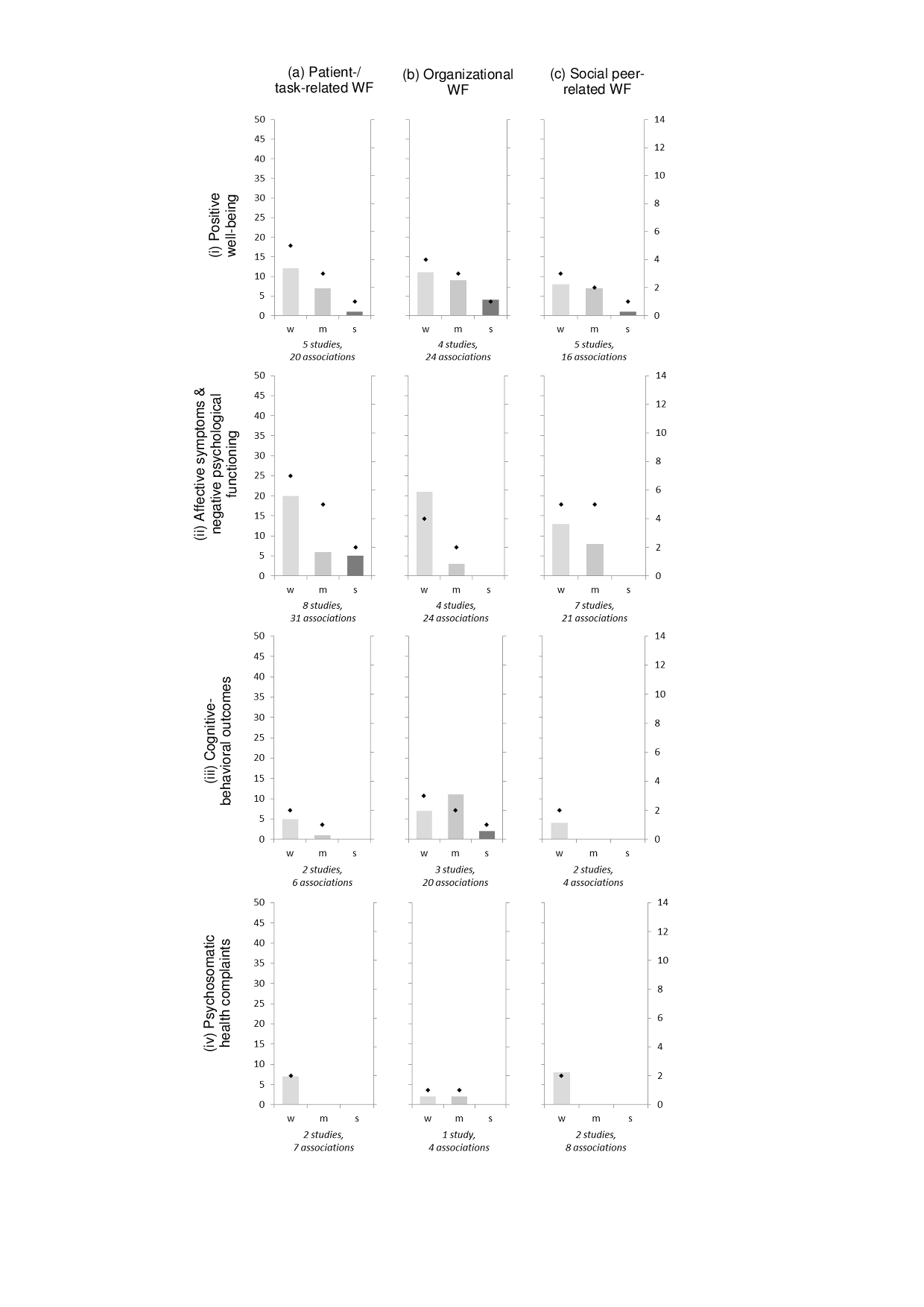

Supplement: S1 Fig — Left axis (bars) denominates frequency of univariate associations; right axis (diamonds) denominates number of original studies describing these relationships; w: weak, m: moderate, s: strong; Text in italics denominates total number of original studies and total number of univariate associations analysing variables out of the respective categories for psychosocial work factors and mental well-being outcomes. (TIF) [file pone.0197375.s001.tif]

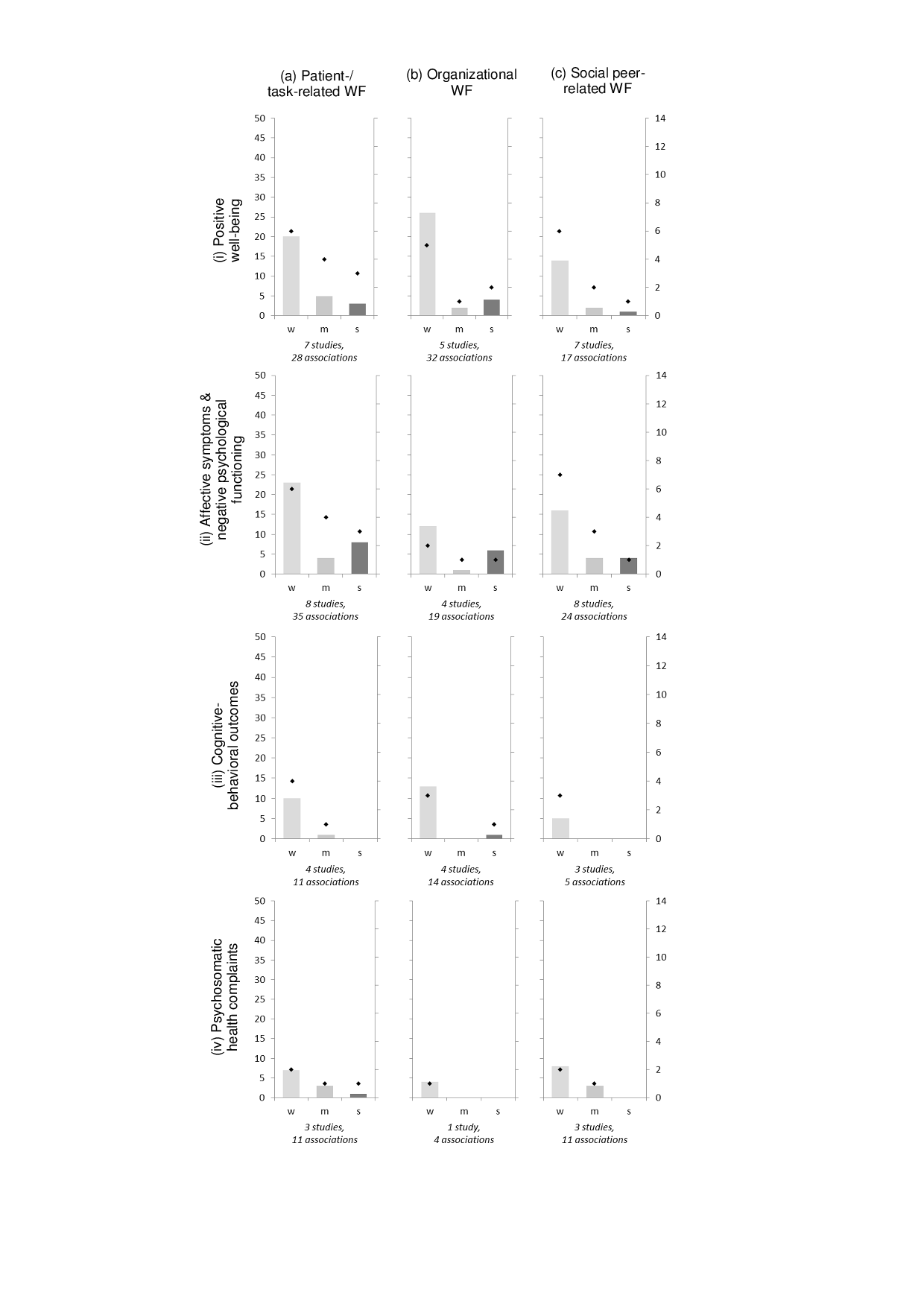

Supplement: S2 Fig — Left axis (bars) denominates frequency of multivariate associations; right axis (diamonds) denominates number of original studies describing these relationships. W: weak, m: moderate, s: strong; Text in italics denominates total number of original studies and total number of multivariate associations analysing variables out of the respective categories for psychosocial work factors and mental well-being outcomes. (TIF) [file pone.0197375.s002.tif]

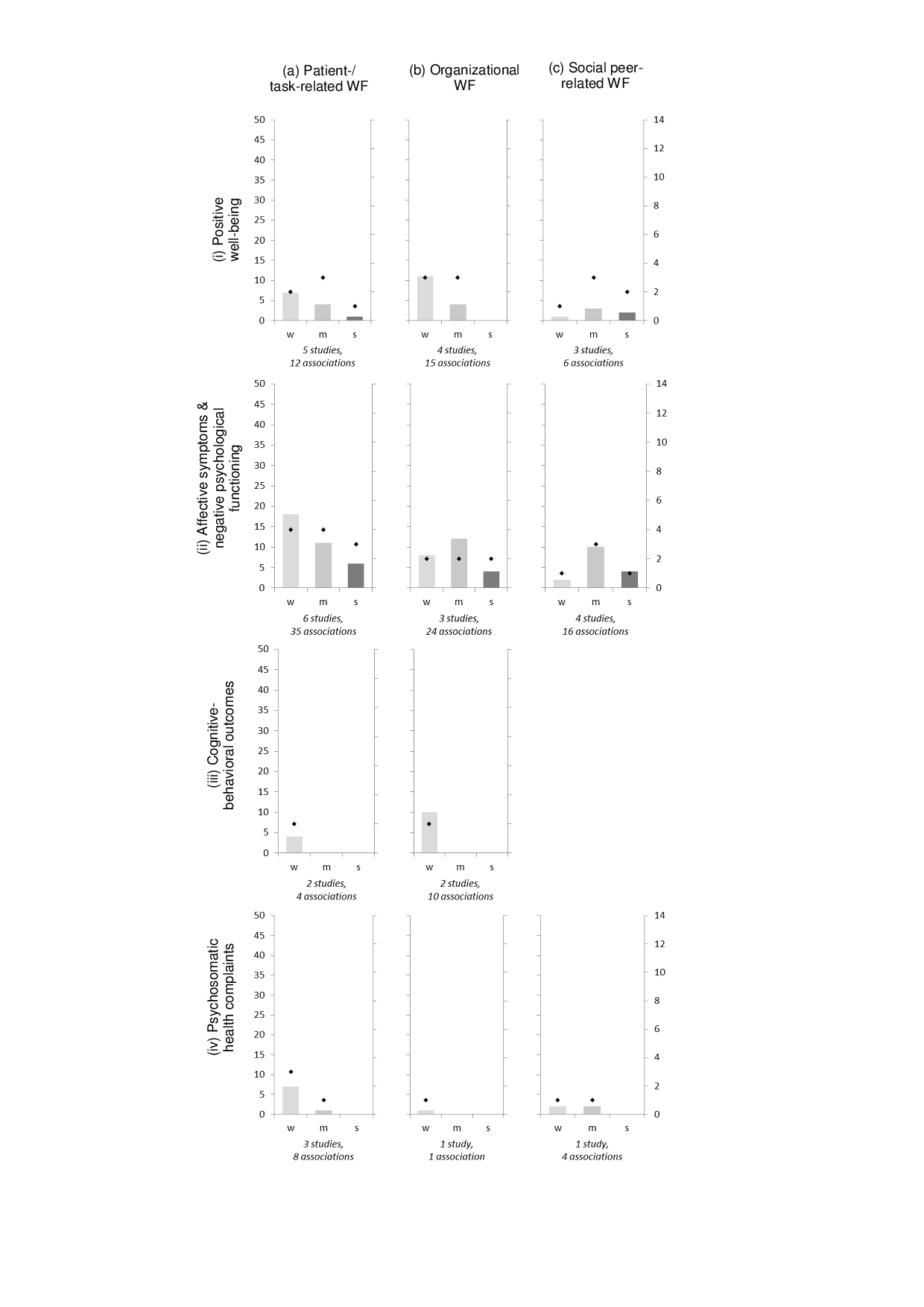

Supplement: S3 Fig — Left axis (bars) denominates frequency of univariate associations; right axis (diamonds) denominates number of original studies describing these relationships; w: weak, m: moderate, s: strong; Text in italics denominates total number of original studies and total number of univariate associations analysing variables out of the respective categories for psychosocial work factors and mental well-being outcomes. (TIF) [file pone.0197375.s003.tif]

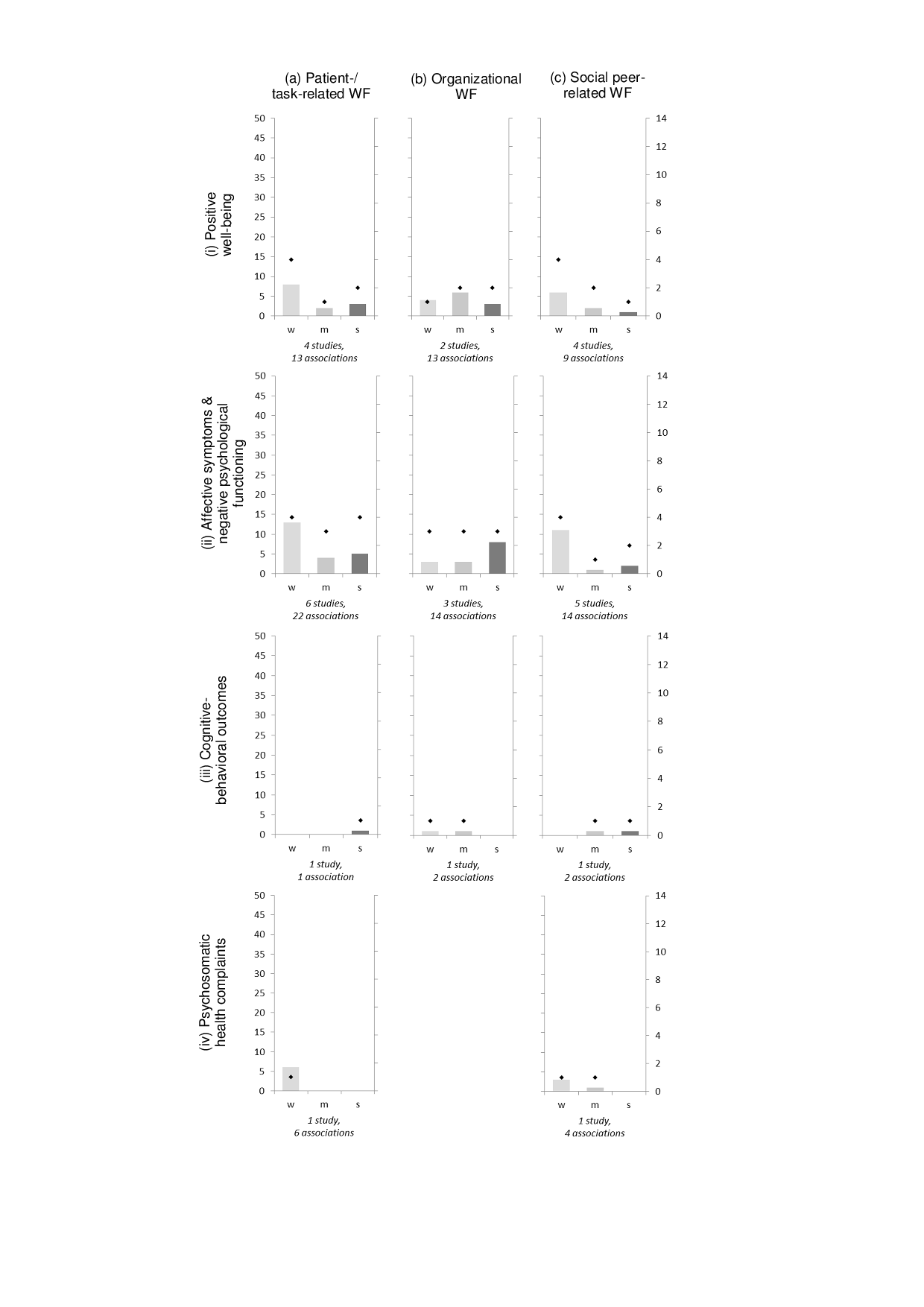

Supplement: S4 Fig — Left axis (bars) denominates frequency of multivariate associations; right axis (diamonds) denominates number of original studies describing these relationships. W: weak, m: moderate, s: strong; Text in italics denominates total number of original studies and total number of multivariate associations analysing variables out of the respective categories for psychosocial work factors and mental well-being outcomes. (TIF) [file pone.0197375.s004.tif]
