# Supplementary material for: Associations between psychosocial work factors and provider mental well-being in emergency departments: A systematic review
Source: PLoS One. 2018 Jun 4;13(6):e0197375. doi: 10.1371/journal.pone.0197375 (PMC5986127; doi:10.1371/journal.pone.0197375)
Supplement: S2 Table — (DOCX) [file pone.0197375.s006.docx]

| Search category | Search term |
| --- | --- |
| Population | (((nurse OR registered nurse OR RN OR nurse practitioner OR physician OR doctor OR intern OR MD OR medic OR clinician OR practitioner OR staff OR crew OR employees OR personnel OR team OR work force OR workers) |
| Setting | AND (emergency room OR emergency medicine OR accident & emergency OR emergency department)) |
| Exposition | AND (work characteristics OR working characteristics OR work environment OR working environment OR psychosocial)) |
| Outcomes | AND (mental health OR mental well being OR mental wellbeing OR burnout OR fatigue OR irritation OR irritability OR depression OR work engagement OR motivation OR job satisfaction OR turnover intention OR work ability) |
