# Supplementary material for: Associations between psychosocial work factors and provider mental well-being in emergency departments: A systematic review
Source: PLoS One. 2018 Jun 4;13(6):e0197375. doi: 10.1371/journal.pone.0197375 (PMC5986127; doi:10.1371/journal.pone.0197375)
Supplement: S7 Table — First author and year of publication in italics; Δ: delta/difference; T2: wave 2; β: standardized regression coefficient/beta; 95%CI: 95% confidence interval; OR: odds ratio; SPC: standardized path coefficient; r: correlation coefficient; SD: standard deviation. (DOCX) [file pone.0197375.s011.docx]

|  | Psychosocial work factors | | |
| --- | --- | --- | --- |
|  | a) Patient- and task-related work factors | b) Organizational work factors | c) Social work factors |
| i) Positive well-being | **Job satisfaction**  Nurses:  *Adriaenssens 2015:*  -Δ Job control:  Job satisfaction T2, β = 0.25  -Δ Job demands:  Job satisfaction T2, β = 0.18  *Adriaenssens 2011:*  -Skill discretion:  Job satisfaction, β = 0.17  -Decision authority:  Job satisfaction, β = 0.13  *Bruyneel 2017:*  -Skill discretion:  Job satisfaction, β = 0.449  *Sawatzky 2012:*  -Competence:  Job satisfaction, β [95%CI] = -0.885 [0.812; 0.965]  Physicians:  *Cydulka 2008:*  -(Problems with) Level of energy needed for work:  High career satisfaction, OR [95%CI] = 0.6 [0.4; 0.9]  -(Problems with) Level of energy needed for work:  Low career satisfaction, OR [95%CI] = 2.2 [1.2; 4.2]  -(Lack of) Exciting work:  High career satisfaction, OR [95%CI] = 5.0 [1.8; 13.6]  -(Lack of) Exciting work:  Low career satisfaction, OR [95%CI] = 4.2 [1.5; 12.0]  -Lack of control over working conditions:  Low career satisfaction, OR [95%CI] = 2.3 [1.0; 5.2]  *Revicki 1997:*  -Role ambiguity:  Work satisfaction, SPC = -0.22  *Taylor 2004:*  -Control of activity mix:  Work satisfaction, r = 0.47  **Work engagement**  Nurses:  *Adriaenssens 2015:*  -Δ Job control:  Work engagement T2, β = 0.21  *Adriaenssens 2011:*  -Skill discretion:  Work engagement, β = 0.32  *Sawatzky 2012*:  -Professional practice:  Engagement, β [95%CI] = 0.173 [0.081; 0.265]  **Feelings of safety**  Nurses:  *Blando 2013*:  -Infrequent verbal abuse:  Feelings of safety, OR [95%CI] = 3.8 [1.6; 9.0]  Mixed sample:  *Gates 2006*:  -Verbal harassment (patients/ visitors):  Feelings of safety, r = -0.338 / r = -0.359  -Sexual harassment (patients/ visitors):  Feelings of safety, r = -0.320/ r = -0.248  -Threats (patients/ visitors):  Feelings of safety, r = -0.324/ r = -0.378  -Assaults (patients/ visitors):  Feelings of safety, r = -0.196/ r = -0.238  **Personal accomplishment**  Nurses:  *Converso 2015*:  -Gratitude from patients:  Personal accomplishment, SPC (SD) = 0.264 (0.122)  -Support from patients:  Personal accomplishment, SPC (SD) = 0.227 (0.114)  -Job autonomy:  Personal accomplishment, SPC (SD) = 0.257 (0.109)  Nurses and physicians:  *Escriba-Aguir 2006*:  -Job control:  Personal accomplishment, OR [95%CI] = 2.55 [1.66; 3.94]  *Wilson 2017*:  -Affected by high mortality:  Personal accomplishment, OR [95%CI] = 0.433 [0.177; 1.060]; adjusted OR [95%CI] = 2.35 [1.12; 3.94]  -Increased load of patients:  Personal accomplishment, OR [95%CI] = 0.950 [0.351; 2.571]  -Infection risk:  Personal accomplishment, OR [95%CI] = 2.458 [0.984; 6.143]; adjusted OR [95%CI] = 1.89 [0.68; 5.27]  **Vitality**  Physicians:  *Escriba-Aguir 2007*:  -Psychological demands:  Vitality, OR [95%CI] = 3.67 [1.87; 7.21]  Nurses:  *Escriba-Aguir 2007*:  -Static physical workload:  Vitality, OR [95%CI] = 2.49 [1.18; 5.22]  **Life satisfaction**  Physicians:  *Taylor 2004*:  -Control of activity mix:  Life satisfaction, r = 0.40  **Well-being/ Happiness**  Physicians:  *Chen 2017*:  -Workload:  Well-being, r = 0.301 | **Job satisfaction**  Nurses:  *Adriaenssens 2011*:  -Rewards:  Job satisfaction, β = 0.25  *Bruyneel 2017*:  -Nursing foundations in quality for care:  Job satisfaction, β = 0.240  -Nurse participation in hospital affairs:  Job satisfaction, β = 0.258  -Nurse management and leadership:  Job satisfaction, β = 0.213  *Lin 2011*:  -Task-oriented leadership:  Nurse satisfaction, SPC = 0.27  -Employee-oriented leadership:  Nurse satisfaction, SPC = 0.30  *Sawatzky 2012*:  -Work overtime:  Job satisfaction, β [95%CI] = 2.147 [1.053; 4.379]  -Staffing resources:  Job satisfaction, β [95%CI] = 1.110 [1.004; 1.227]  Physicians:  *Clem 2008*:  -Compensation:  Career satisfaction, OR [95%CI] = 2.18 [1.42; 3.36]  -Career advancement:  Career satisfaction, OR [95%CI] = 4.27 [2.66; 6.87]  -Schedule flexibility:  Career satisfaction: OR [95%CI] = 4.17 [2.78; 6.27]  *Cydulka 2008*:  -(Problems with) Enough time for personal life:  High career satisfaction, OR [95%CI] = 0.5 [0.3; 0.9]  -(Problems with) Enough time for personal life:  Low career satisfaction, OR [95%CI] = 2.8 [1.2; 6.3]  -(Problems with) Hospital administration:  High career satisfaction, OR [95%CI] = 0.5 [0.2; 0.9]  -(Problems with) Length of shifts:  High career satisfaction, OR [95%CI] = 0.3 [0.1; 0.5]  -(Problems with) Subspecialty support:  High career satisfaction, OR [95%CI] = 0.5 [0.3; 0.9]  -Fair compensation:  High career satisfaction, OR [95%CI] = 2.5 [1.1]  -(Lack of) Job security:  High career satisfaction, OR [95%CI] = 2.1 [1.1; 4.2]  -(Lack of) Job security:  Low career satisfaction, OR [95%CI] = 2.7 [1.2; 6.3]  -(Lack of) Personal reward:  High career satisfaction, OR [95%CI] = 2.8 [1.2; 6.7]  -(Lack of) Personal reward:  Low career satisfaction, OR [95%CI] = 4.7 [1.8; 12.2]  *Taylor 2004*:  -Control of hours worked:  Work satisfaction, r = 0.40  **Work engagement**  Nurses:  *Adriaenssens 2015*:  -Δ Reward:  Work engagement T2, β = 0.14  *Adriaenssens 2011*:  -Rewards:  Work engagement, β = 0.13  -Work procedures:  Work engagement, β = 0.18  *Sawatzky 2012*:  -Staffing resources:  Engagement, β [95%CI] = 0.270 [0.105; 0.436]  -Nursing management:  Engagement, β [95%CI] = 0.367 [0.214; 0.520]  **Feelings of safety**  Nurses:  *Blando 2013*:  -Adequate security response time:  Feelings of safety, OR [95%CI] = 5.4 [2.0; 15.1]  -Adequate security equipment:  Feelings of safety, OR [95%CI] = 3.8 [1.5; 9.4]  **Personal accomplishment**  Physicians:  *Jalili 2013*:  -Physical work environment:  Personal accomplishment, r = -0.205  -Economic problems and future of EM as a career:  Personal accomplishment, r = -0.276  -Difficulties to balance professional and private life:  Personal accomplishment, r = -0.275  -Educational issues (morning reports, progress notes, educational rounds etc.):  Personal accomplishment, r = -0.339  -Image of emergency medicine in media:  Personal accomplishment, r = -0.248  -Consultant unavailability:  Personal accomplishment, r = -0.226  **Compassion satisfaction**  Nurses:  *Sawatzky 2012*:  -Staffing resources:  Compassion satisfaction, β [95%CI] = -0.304 [-0.537; -0.071]  **Life satisfaction**  Physicians:  *Taylor 2004*:  -Control of hours worked:  Life satisfaction, r = 0.41  **Well-being/ Happiness**  Physicians:  *Chen 2017*:  -Emergency safety:  Well-being, r = 0.301  -Salary and benefit:  Well-being, r = 0.295 | **Job satisfaction**  Nurses:  *Adriaenssens 2015*:  -Δ Social support:  Job satisfaction T2, β = 0.22  *Adriaenssens 2011*:  -Social support from colleagues:  Job satisfaction, β = 0.16  *Bruyneel 2017*:  -Social support supervisor:  Job satisfaction, β = 0.210  Physicians:  *Clem 2008*:  -Relationship with colleagues:  Career satisfaction, OR [95%CI] = 1.77 [1.08; 2.88]  *Revicki 1997*:  -Work-group support:  Work satisfaction, SPC = 0.35  *Somville 2016*:  -Colleagues support:  Job satisfaction, β = 0.33  **Work engagement**  Nurses:  *Adriaenssens 2011*:  -Social support from supervisor:  Work engagement, β = 0.17  *Sawatzky 2012*:  -Collaboration with physicians:  Engagement, β [95%CI] = 0.307 [0.123; 0.493]  **Personal accomplishment**  Nurses:  *Garcia-Izquierdo 2012*:  -Interpersonal conflicts:  Professional efficacy, β = -0.209  -Lack of social support:  Professional efficacy, β = -0.163  Physicians and nurses:  *Wilson 2017*:  -More criticism:  Personal accomplishment, OR [95%CI] = 0.910 [0.376; 2.201]  -Departmental activities for staff bonding:  Personal accomplishment, OR [95%CI] = 0.703 [0.287; 1.718]  **Vitality**  Physicians:  *Escriba-Aguir 2007*:  -Social support colleagues:  Vitality, OR [95%CI] = 3.19 [1.65; 6.14]  **Compassion satisfaction**  Nurses:  *Hunsaker 2015*:  -Support from manager:  Compassion satisfaction, β = 0.292  **Resilience**  Nurses:  *Hsieh 2016*:  -Peer support:  Personal strength (resilience), r = 0.31  -Peer support:  Family cohesion (resilience), r = 0.38  -Peer support:  Social resource (resilience), r = 0.44  -Peer support:  Social competence (resilience), r = 0.32  -Peer support:  Structured style (resilience), r = 0.34 |
| ii) Affective symptoms and negative psychological functioning | **Emotional exhaustion**  Nurses:  *Adriaenssens 2015*:  -Δ Job demands:  Emotional exhaustion T2, β = -0.17  *Bruyneel 2017*:  -Skill discretion:  Emotional exhaustion: β = -3.527  -Work/time demands:  Emotional exhaustion, β = -7.159  -Decision authority:  Emotional exhaustion, β = -2.760  -Physical demands:  Emotional exhaustion, β = -2.894  *Converso 2015*:  - Psychological demands:  Emotional exhaustion, SPC (SD) = 0.288 (0.126)  -Gratitude from patients:  Emotional exhaustion, SPC (SD) = -0.335 (0.147)  *Escriba-Aguir 2007*:  -Psychological demands:  Emotional exhaustion (nurses), OR [95%CI] = 4.98 [2.04; 12.17]  *Garcia-Izquierdo 2012*:  -Excessive workload:  Emotional exhaustion, β = 0.317  *O’Mahony 2011*:  -Enough time to discuss patient care:  Emotional exhaustion, τ = -0.198  Physicians:  *Escriba-Aguir 2007*:  -Psychological demands:  Emotional exhaustion, OR [95%CI] = 5.66 [2.73; 11.69]  *Jalili 2013*:  -Work overload:  Emotional exhaustion, OR = 3.1  *Toker 2015*:  -Appreciation by patients and relatives– Emotional exhaustion  -Exposure to violence–  Emotional exhaustion  Nurses and physicians:  *Escriba-Aguir 2006*:  -Psychological-emotional demands:  Emotional exhaustion, OR [95%CI] = 4.66 [2.75; 2.63]  -Job control:  Emotional exhaustion, OR [95%CI] = 1.65 [1.04; 2.63]  *Weigl 2017*:  -Time pressure:  Emotional exhaustion, β [95%CI] = 0.52 [0.26; 0.79]  *Wilson 2017*:  -Affected by high mortality:  Emotional exhaustion, OR [95%CI] = 2.244 [0.892; 5.648]; adjusted OR [95%CI] = 1.47 [0.53; 4.04]  -Increased load of patients:  Emotional exhaustion, OR [95%CI] = 3.182 [1.287; 7.867]; adjusted OR [95%CI] = 2.12 [0.76; 5.92]  -Infection risk:  Emotional exhaustion, OR [95%CI] = 0.889 [0.364; 2.172]  **Depersonalization**  Nurses:  *Garcia-Izquierdo 2012*:  -Excessive workload:  Cynicism, β = 0.170  *O’Mahony 2011*:  -Enough time to discuss patient care:  Depersonalization, τ = -0.228  Physicians:  *Toker 2015*:  -Appreciation by patients and relatives–Depersonalization  -Exposure to violence-  Depersonalization  Physicians and nurses:  *Wilson 2017*:  -Affected by high mortality[[56](#_ENREF_56)]:  Depersonalization, OR [95%CI] = 1.848 [0.748; 4.868]  -Increased load of patients:  Depersonalization, OR [95%CI] = 4.173 [1.640; 10.617]; adjusted OR [95%CI] = 1.84 [0.60; 5.65]  -Infection risk:  Depersonalization, OR [95%CI] = 0.688 [0.259; 1.830]  **Burnout**  Physicians:  *Ben-Itzhak 2015*:  -Meaningful job:  Burnout, β [95%CI] = -3.144 [-6.24; -0.045]  *Cydulka 2008*:  -(Problems with) Level of energy needed for work:  Burnout, OR [95%CI] = 3.0 [2.0; 4.7]  -Lack of control over working conditions:  Burnout, OR [95%CI] = 1.9 [1.1; 3.4]  -Problems with knowing enough:  Burnout, OR [95%CI] = 2.0 [1.2; 3.2]  -Level of patient acuity:  Burnout, OR [95%CI] = 2.3 [1.2; 4.2]  ED workers:  *Hamdan 2017*:  -Exposure to physical violence:  High burnout, OR [95%CI] = 2.017 [1.121; 3.631]  -Exposure to non-physical violence:  High burnout, OR [95%CI] = 1.792 [0.868; 3.697]  **PTSD**  Nurses:  *Adriaenssens 2012*:  -Frequency of exposure to traumatic events:  Post-traumatic stress reactions, β = 0.23  Physicians:  *Somville 2016*:  -Traumatic events:  Posttraumatic stress reactions, β = 0.23  **Reliving experience**  Physicians:  *Zahid 1999*:  -Mild violence:  Reliving experience, r = 0.265  **Fearfulness**  Physicians:  *Zahid 1999*:  -Mild violence:  Fearfulness, r = 0.334  -Severe violence:  Fearfulness, r = 0.230  **Psychological distress**  Nurses:  *Adriaenssens 2012*:  -Frequency of exposure to traumatic event:  Psychological distress, β = 0.15  Physicians:  *Somville 2016*:  -Traumatic events:  Psychological distress, β = 0.14  -Occurrence violence:  Psychological distress, β = 0.15  **Psychosomatic distress**  Nurses:  *Adriaenssens 2011*:  -Work/time demands:  Psychosomatic distress, β = -0.31  **Anxiety**  Physicians:  *Taylor 2004*:  -Control of activity mix:  Anxiety, r = -0.29  **Mental health**  Physicians:  *Escriba-Aguir 2007*:  -Psychological demands:  Mental health, OR [95%CI] = 4.01 [1.94; 8.31]  Nurses:  *Escriba-Aguir 2007*:  -Psychological demands:  Mental health, OR [95%CI] = 2.34 [1.06; 5.17]  **Depression**  Physicians:  *Revicki 1997*:  -Role ambiguity:  Depression, SPC = 0.13  *Taylor 2004*:  -Control of activity mix:  Depression, r = -0.29  *Zahid 1999*:  -Mild violence:  Depression, r = 0.457  **Work stress**  Physicians:  *Revicki 1997*:  -Role ambiguity:  Work stress, SPC = 0.42  *Taylor 2004*:  -Control of activity mix:  Work stress, r = -0.28  -Control of activity mix:  Total stress, r = -0.37  Nurses:  *Wu 2012*:  -Role overload:  Occupational stress, β = 0.407  -Role boundary:  Occupational stress, β = 0.283  -Role insufficiency:  Occupational stress, β = 0.261  **Irritation**  Nurses and physicians:  *Weigl 2017*:  -Time pressure:  Irritation, β [95%CI] = 0.48 [0.24; 0.73]  **Nervousness**  Nurses:  *Crilly 2017*:  -Self-realisation:  Nervousness, r = 0.60  -Workload:  Nervousness, r = 0.69 | **Emotional exhaustion**  Nurses:  *Bruyneel 2017*:  -Nurse staffing:  Emotional exhaustion, β = -2.897  *O’Mahony 2011*:  -Quality assurance program:  Emotional exhaustion, τ = -0.256  -Administration consults:  Emotional exhaustion, τ = -0.229  -Non-punitive management:  Emotional exhaustion, τ = -0.209  -High standards are expected by administration:  Emotional exhaustion, τ = -0.228  -Administration listens and responds:  Emotional exhaustion, τ = -0.337  Physicians:  *Jalili 2013*:  -Economic problems and future career in emergency medicine:  Emotional exhaustion, OR = 2.28  -Difficulties to balance professional and private life:  Emotional exhaustion, OR = 9.2  -New information and technologies (internet, etc.):  Emotional exhaustion, OR = 0.56  *Toker 2015*:  -Presence of consultant–  Emotional exhaustion  **Depersonalization**  Nurses:  *Garcia-Izquierdo 2012*:  -Lack of resources:  Cynicism, r = 0.182  Physicians:  *Jalili 2013*:  -Shortage of equipment:  Depersonalization, r = 0.230  -Physical environment at work:  Depersonalization, r = 0.310  -Problems in relationship with other services:  Depersonalization, r = 0.294  -Economic problems and future of EM as a career:  Depersonalization, r = 0.363  -Difficulties to balance professional and private life:  Depersonalization, r = 0.417  -Educational issues (morning reports, progress notes, educational rounds etc.):  Depersonalization, r = 0.432  -Image of emergency medicine in media:  Depersonalization, r = 0.464  - Consultant unavailability:  Depersonalization, r = 0.259  -New information and technologies (internet, etc.):  Depersonalization, r = 0.287  **Burnout**  Physicians:  *Cydulka 2008*:  -(Problems with) Enough time for personal life:  Burnout, OR [95%CI] = 1.9 [1.1; 3.2]  -(Problems with) Length of shifts:  Burnout, OR [95%CI] = 3.7 [2.0; 6.9]  -(Lack of) Personal reward:  Burnout, OR [95%CI] = 2.8 [1.2; 6.4]  -Problems with number of night shifts:  Burnout, OR [95%CI] = 3.6 [2.0; 6.2]  -Problems with opportunity to attend conferences:  Burnout, OR [95%CI] = 3.0 [1.5; 5.8]  *Estryn-Behar 2011*:  -Work/family conflict (medium/high):  Burnout, OR [95%CI] = 2.36 [1.11; 5.05], OR [95%CI] = 6.14 [2.89; 13.04]  Nurses:  *Sawatzly 2012*:  -Staffing resources:  Burnout, β [95%CI] = -0.305 [-0.511; -0.099]  **Psychosomatic distress**  Nurses:  *Adriaenssens 2015*:  -Δ Material resources:  Psychosomatic distress T2, β = -0.17  **Anxiety**  Physicians:  *Taylor 2004*:  -Control of hours worked:  Anxiety, r = -0.27  **Depression**  Physicians:  *Taylor 2004*:  -Control of hours worked:  Depression, r = -0.27  **Work stress**  Physicians:  *Taylor 2004*:  -Control of hours worked:  Work stress, r = -0.23  -Control of hours worked:  Total stress, r = -0.34 | **Emotional exhaustion**  Nurses:  *Adriaenssens 2015*:  -Δ Social support:  Emotional exhaustion T2, β = -0.24  -Δ Social harassment:  Emotional exhaustion T2, β = -0.14  *Bruyneel 2017*:  -Social support supervisor:  Emotional exhaustion, β = -1.554  -Collegial nurse-physician relations:  Emotional exhaustion, β = -4.916  *Escriba-Aguir 2007*:  -Social support supervisor:  Emotional exhaustion (nurses), OR [95%CI] = 2.89 [1.29; 6.51]  *Garcia-Izquierdo 2012*:  -Lack of emotional support:  Emotional exhaustion, β = 0.241  *O’Mahony 2011*:  -Nurse physician collaboration:  Emotional exhaustion, τ = -0.205  Physicians:  *Escriba-Aguir 2007*:  -Social support colleagues:  Emotional exhaustion, OR [95%CI] = 2.17 [1.10; 4.26]  *Toker 2015*:  -Appreciation by supervisor and coworkers–  Emotional exhaustion  -Compliance with personnel – Emotional exhaustion  Nurses and physicians:  *Escriba-Aguir 2006*:  -Social support supervisor:  Emotional exhaustion, OR [95%CI] = 1.64 [1.04; 2.59]  *Wilson 2017*:  -More criticism:  Emotional exhaustion, OR [95%CI] = 1.935 [0.857; 4.369]; adjusted OR [95%CI] = 1.29 [0.51; 3.27]  -Departmental activities for staff bonding:  Emotional exhaustion, OR [95%CI] = 2.568 [1.129; 5.841]; adjusted OR [95%CI] = 1.84 [0.75; 4.54]  **Depersonalization**  Nurses:  *Garcia-Izquierdo 2012*:  -Lack of emotional support:  Cynicism, β = 0.233  -Interpersonal conflicts:  Cynicism, β = 0.239  *O’Mahony 2011*:  -Nurse physician collaboration:  Depersonalization, τ = -0.214  -Teamwork:  Depersonalization, τ = -0.294  Physicians:  *Toker 2015*:  -Compliance with personnel–Depersonalization  -Appreciation by supervisor and coworkers–  Depersonalization  Physicians and nurses:  *Wilson 2017*:  -More criticism:  Depersonalization, OR [95%CI] = 4.522 [1.831; 11.166]; adjusted OR [95%CI] = 3.57 [1.25; 10.19]  -Departmental activities for staff bonding:  Depersonalization, OR [95%CI] = 3.455 [1.427; 8.364]; adjusted OR [95%CI] = 2.19 [0.78; 6.11]  **Burnout**  Physicians:  *Cydulka 2008*:  - Problems with colleagues:  Burnout, OR [95%CI] = 3.7 [1.8; 8.0]  Nurses:  *Hunsaker 2015*:  -Support from manager:  Burnout, β = -0.373  **PTSD**  Nurses:  *Adriaenssens 2012*:  -Social support supervisor:  Post-traumatic stress reactions, β = -0.16  **Psychological distress**  Nurses:  *Adriaenssens 2012*:  -Social support supervisor:  Psychological distress, β = 0.-24  **Psychosomatic distress**  Nurses:  *Adriaenssens 2015*:  -Δ Social harassment:  Psychosomatic distress T2, β = -0.17  *Adriaenssens 2011*:  -Social support from supervisor:  Psychosomatic distress, β = -0.27  **Mental health**  Physicians:  *Escriba-Aguir 2007*:  -Social support supervisor:  Mental health, OR [95%CI] = 1.88 [1.03; 3.44]  -Social support colleagues:  Mental health, OR [95%CI] = 3.29 [1.68; 4.78]  **Depression**  Nurses:  *Hsieh 2016*:  -Peer support:  Depression, r = -0.26  Physicians:  *Revicki 1997*:  -Peer support:  Depression, SPC = -0.06  -Work-group support:  Depression, SPC = -0.01  **Work stress**  Physicians:  *Revicki 1997*:  -Peer support:  Work stress, SPC = -0.14  -Work-group support:  Work stress, SPC = -0.20  **Irritation**  Nurses and physicians:  *Weigl 2017*:  -Supervisor support:  Irritation, β [95%CI] = -0.24 [-0.47; -0.02]  **Nervousness**  Nurses:  *Crilly 2017*:  -Conflict:  Nervousness, r = 0.38  **Compassion fatigue**  Nurses:  *Hunsaker 2015*:  -Support from manager:  Compassion fatigue, β = -0.230 |
| iii) Cognitive-behavioural outcomes | **Turnover intention/ Intention to leave**  Nurses:  *Adriaenssens 2011*:  -Skill discretion:  Turnover intention, β = -0.20  *Bruyneel 2017*:  -Skill discretion:  Turnover intention: β = -0.318  -Decision authority:  Turnover intentions, β = -0.223  *Sawatzky 2012*:  -Professional practice:  Intention to leave nursing, OR [95%CI] = 1.058 [1.002; 1.117]  Physicians:  *Chen 2017*:  -Workload:  Turnover intention, r = 0.061  *Estryn-Behar 2011*:  -Influence at work (female physicians):  Intention to leave, OR [95%CI] = 3.44 [1.29; 9.18]  ED workers:  *Hamdan 2015*:  -Exposure to violence (general):  Intention to quit work in ED, OR [95%CI] = 3.48 [1.879; 6.433]  -Exposure to physical violence:  Intention to quit work in ED, OR [95%CI] = 2.18 [1.21; 3.90]  -Exposure to non-physical violence:  Intention to quit work in ED, OR [95%CI] = 3.17 [1.78; 5.67] | **Turnover intention/ Intention to leave**  Nurses:  *Adriaenssens 2015*:  -Δ Work agreements:  Turnover intention T2, β = -0.22  *Adriaenssens 2011*:  -Rewards:  Turnover intention, β = -0.16  *Bruyneel 2017*:  -Nurse staffing:  Turnover intention, β = -0.160  -Career development and opportunities:  Turnover intentions, β = -0.189  Physicians:  *Chen 2017*:  -Emergency safety:  Turnover intention, r = 0.121  -Salary and benefit:  Turnover intention, r = 0.143  *Lin 2012*:  -Clan culture:  Intent to leave, β = -0.28  -Market culture:  Intent to leave, β = 0.27  **Unit performance**  Nurses and physicians:  *Lin 2011*:  -Task-oriented leadership:  Unit performance, SPC = 0.58  **Extra-role behaviour**  Physicians:  *Williams 2007*:  -Entrepreneurial culture:  Extra-role behaviour, SPC = 0.44  **Patient commitment**  Physicians:  *Williams 2007*:  -Bureaucratic culture:  Patient commitment, SPC = 0.18  **Affective commitment**  Nurses:  *Young-Ritchie 2009*:  - Structural empowerment:  Affective commitment, SPC = .61 | **Turnover intention/ Intention to leave**  Nurses:  *Bruyneel 2017*:  -Social support supervisor:  Turnover intention, β = -0.146  Physicians:  *Estryn-Behar 2011*:  -Harassment by superiors:  Intention to leave, OR [95%CI] = 2.89 [1.61; 5.18] |
| iv) Health complaints | **Somatic complaints**  Nurses:  *Adriaenssens 2012*:  -Frequency of exposure to traumatic event:  Somatic complaints, β = 0.17  Physicians:  *Somville 2016*:  -Occurrence violence:  Somatic complaints, β = 0.15  **Physical symptoms**  Physicians:  *Taylor 2004*:  -Control of activity mix:  Physical symptoms, r = -0.15  **Sleep problems**  Nurses:  *Adriaenssens 2012*:  -Frequency of exposure to traumatic event:  Sleep problems, β = 0.16  Physicians:  *Zahid 1999*:  -Severe violence:  Sleeplessness, r = 0.203  **Fatigue**  Nurses:  *Adriaenssens 2011*:  -Work/time demands:  Fatigue, β = -0.21  Physicians:  *Somville 2016*:  -Occurrence violence:  Fatigue, β = 0.27  **Physical quality of life**  Nurses:  *Kogien 2014*:  -Intellectual discernment:  Physical domain QoL (general score), OR [95%CI] = 2.94 [1.34; 6.41]  -Intellectual discernment:  Facet energy and fatigue, OR [95%CI] = 2.62 [1.21; 5.68]  -Intellectual discernment:  Facet mobility, OR [95%CI] = 3.89 [1.77; 8.56]  -Intellectual discernment:  Facet daily life activities, OR [95%CI] = 2.63 [1.22; 5.67] | **Physical symptoms**  Physicians:  *Taylor 2004*:  -Control of hours worked:  Physical symptoms, r = -0.16  **Fatigue**  Nurses:  *Adriaenssens 2011*:  -Rewards:  Fatigue, β = -0.17  -Work procedures:  Fatigue, β = -0.17 | **Somatic complaints**  Nurses:  *Adriaenssens 2012*:  -Social support supervisor:  Somatic complaints, β = -0.17  Physicians:  *Somville 2016*:  -Colleagues support:  Somatic complaints, β = -0.36  -Supervisor support:  Somatic complaints, β = 0.22  **Sleep problems**  Nurses:  *Adriaenssens 2012*:  -Social support supervisor:  Sleep problems, r = -0.20  -Social support colleagues:  Sleep problems, r = -0.13  **Fatigue**  Nurses:  *Adriaenssens 2012*:  -Social support supervisor:  Fatigue, β = -0.13  -Social support colleagues:  Fatigue, β = -0.13  Physicians:  *Somville 2016*:  -Colleagues support:  Fatigue, β = -0.23  **Physical quality of life**  Nurses:  *Kogien 2014*:  -Social support:  Physical domain QoL (general score), OR [95%CI] = 2.81 [1.43; 5.53]  -Social support:  Facet mobility, OR [95%CI] = 2.29 [1.12; 4.67]  -Social support:  Facet sleep and rest, OR [95%CI] = 2.95 [1.46; 5.94] |
